# An Unbiased Approach to Identifying Cellular Reprogramming-Inducible Enhancers

**DOI:** 10.3390/ijms252313128

**Published:** 2024-12-06

**Authors:** Eleftheria Klagkou, Dimitrios Valakos, Spyros Foutadakis, Alexander Polyzos, Angeliki Papadopoulou, Giannis Vatsellas, Dimitris Thanos

**Affiliations:** 1 Biomedical Research Foundation, Academy of Athens (BRFAA), 4 Soranou Efesiou St., 11527 Athens, Greece; eklagou@bioacademy.gr (E.K.); dvalakos@bioacademy.gr (D.V.); foutadakiss@gmail.com (S.F.); appolyzos@gmail.com (A.P.); angeliki.papadopoulou@unil.ch (A.P.); gvatsellas@bioacademy.gr (G.V.); 2 Section of Biochemistry and Molecular Biology, Department of Biology, School of Science, National and Kapodistrian University of Athens (NKUA), Panepistimiopolis, Zografou, 15772 Athens, Greece; 3 Hellenic Institute for the Study of Sepsis (HISS), 11528 Athens, Greece; 4 Sanford I. Weill Department of Medicine, Sandra and Edward Meyer Cancer Center, Weill, Cornell Medicine, New York, NY 10065, USA; 5 Department of Computational Biology, University of Lausanne, 1015 Lausanne, Switzerland; 6 Department of Biology, School of Sciences and Engineering, University of Crete, 70013 Irakleio, Greece

**Keywords:** transcriptional regulation, transcription factors, enhancers, chromatin structure, genomics, cellular reprogramming, OSKM, iPSCs

## Abstract

Cellular reprogramming of somatic cells towards induced pluripotency is a multistep stochastic process mediated by the transcription factors Oct4, Sox2, Klf4 and c-Myc (OSKM), which orchestrate global epigenetic and transcriptional changes. We performed a large-scale analysis of integrated ChIP-seq, ATAC-seq and RNA-seq data and revealed the spatiotemporal highly dynamic pattern of OSKM DNA binding during reprogramming. We found that OSKM show distinct temporal patterns of binding to different classes of pluripotency-related enhancers. Genes involved in reprogramming are regulated by the coordinated activity of multiple enhancers, which are sequentially bound by OSKM for strict transcriptional control. Based on these findings, we developed an unbiased approach to identify Reprogramming-Inducible Enhancers (RIEs), constructed enhancer-traps and isolated cells undergoing reprogramming in real time. We used a representative RIE taken from the *Upp1* gene fused to *Gfp* and isolated cells at different time-points during reprogramming and found that they have unique developmental capacities as they are reprogrammed with high efficiency due to their distinct molecular signatures. In conclusion, our experiments have led to the development of an unbiased method to identify and isolate reprogrammable cells in real time by exploiting the functional dynamics of OSKM, which can be used as efficient reprogramming biomarkers.

## 1. Introduction

Cellular reprogramming of somatic cells towards induced Pluripotent Stem Cells (iPSCs) via the over-expression of *Oct4*, *Sox2*, *Klf4* and *c-Myc* (OSKM) provides an excellent model system to study the mechanisms by which a small set of transcription factors (TFs) could reactivate the pluripotency gene network to drive cell fate specification [1,2]. Reprogramming is a stochastic process, achieved only in a few cells that manage to alter their chromatin structure to efficiently and appropriately regulate the necessary gene modules [3,4,5]. During the early stages of reprogramming, somatic cells need to undergo a series of morphological and functional changes, including, among others, an increase in the proliferation rate, a transition towards glycolytic metabolic pathways and a decrease in size [4,6]. In addition, mesenchymal cells undergo the Mesenchymal to Epithelial Transition process (MET) between day 2 and day 4 of reprogramming [7,8,9]. We have previously identified a gene regulatory network (GRN) reconstructed from nine transcriptional regulators (TRs), the 9TR-GRN, which is required for the establishment of pluripotency [10]. The 9TR-GRN consists of *Taf1c*, *Tead4*, *Tfap4*, *Rcan1*, *Cbfa2t3*, *Gli2*, *Irf6*, *Ovol1* and the *Nanog* TFs, all of which are direct OSKM targets. The 9TR-GRN is assembled in a stochastic and stepwise manner between days 1 and 6 of reprogramming only in a small number of cells expressing all factors, and is required for the gradual establishment of pluripotency [10]. These and other studies have suggested that OSKM-induced cellular reprogramming is highly plastic, as it is accompanied by the transient expression of genes from unrelated lineages, regardless of the starting cell type or species [11].

Previous landmark studies have provided insights for the OSKM-driven epigenetic and transcriptional changes occurring in the cells undergoing reprogramming [12,13,14,15,16,17,18,19,20,21]. Oct4, Sox2 and Klf4 function as pioneer transcription factors by binding to Mouse Embryonic Fibroblast (MEF)-closed chromatin during the first days of reprogramming with or without c-Myc [14,18,19,22,23]. OSKM trigger chromatin opening in these sites leading to the stepwise induction of pluripotency genes either alone or in combination with other transcriptional regulators [12,13,14,15,17,18,19,20,21,24,25]. In parallel, OSKM participate in the deactivation of somatic and mesenchymal gene networks regulated by the presence of macroH2A-containing nucleosomes [26]. In addition, OSKM bind accessible and active somatic enhancers causing repression of their associated genes through various complex mechanisms including interactions with the somatic factors, whereas their concomitant binding to pluripotency enhancers activates the expression of the linked genes. OSKM-driven somatic gene repression involves chromatin deacetylation, displacement and/or down-regulation of the somatic TFs and up-regulation of negative transcriptional regulators [17,18,19,24].

Given that cellular reprogramming is a purely stochastic process, predictions regarding which cells will be reprogrammed and the timing for completion of the process are currently impossible. However, various gene markers have been proposed for the identification of pure and efficiently reprogrammable cell populations in vivo [27]. Among them, the best markers for mouse cells involve the early down-regulation of the mesenchymal surface antigen Thy1, the activated expression of alkaline phosphatase (*Alpl*, AP), the up-regulation of the embryonic antigen SSEA1, and the late activation of the *Oct4* and/or the *Nanog* regulatory regions and the reconstruction of the 9TR-GRN [10,28,29,30,31,32]. Despite the existing methods to study cell populations entering the efficient reprogramming path(s), identifying the actual mechanisms responsible for the gradual acquisition of pluripotency still remains elusive. Given the molecular complexity of the reprogramming process and the development of various reprogramming systems (different TF combinations, chemical molecules, genetically modified cells, etc.) [27,33,34,35,36], it will be beneficial to establish new, efficient ways to isolate cells during their transition to pluripotency in real time, in order to illuminate the shady processes controlling reprogramming.

In this study, we performed a large-scale analysis of integrated ChIP-seq, ATAC-seq and RNA-seq data and revealed the spatiotemporal highly dynamic and complex pattern of OSKM binding to the mouse genome during the course of cellular reprogramming. The combinatorial integration of multiple datasets ensures a more reliable and complete picture of the genomic OSKM binding activity, free of putative system-dependent discrepancies. We found that OSKM show distinct temporal patterns of binding at different groups of Embryonic Stem Cells (ESC) enhancers. These highly dynamic and complex DNA binding events are associated with the function and expression of the neighboring genes by affecting the local chromatin dynamics. We found that genes essential for reprogramming (MET/EMT and pluripotency genes) are regulated by the coordinated activity of multiple enhancers, which are sequentially bound by OSKM to warrant strict transcriptional control. Based on these findings, we developed an unbiased approach to identify Reprogramming-Inducible Enhancers (RIEs), aiming to generate enhancer-traps for isolating in real-time cells undergoing reprogramming. We used a construct bearing the RIE taken from the *Upp1* gene fused to *Gfp* as a marker to isolate cells undergoing reprogramming in real time. We demonstrated that these cells have acquired unique developmental capacities since they are reprogrammed with high efficiency due to their distinct molecular signatures, such as the high-level expression of 9TR-GRN components, which are co-expressed in early iPSCs cells. In conclusion, our experiments have led to the development of an unbiased method to identify and isolate reprogrammable cells in real time by exploiting the functional dynamics of OSKM that controls the transcriptional potential of RIEs, which can be used as efficient reprogramming biomarkers.

## 2. Results

### 2.1. Creation of an Integrated ChIP-Seq Dataset to Monitor Global Binding of OSKM During Cellular Reprogramming

**Figure 1:**
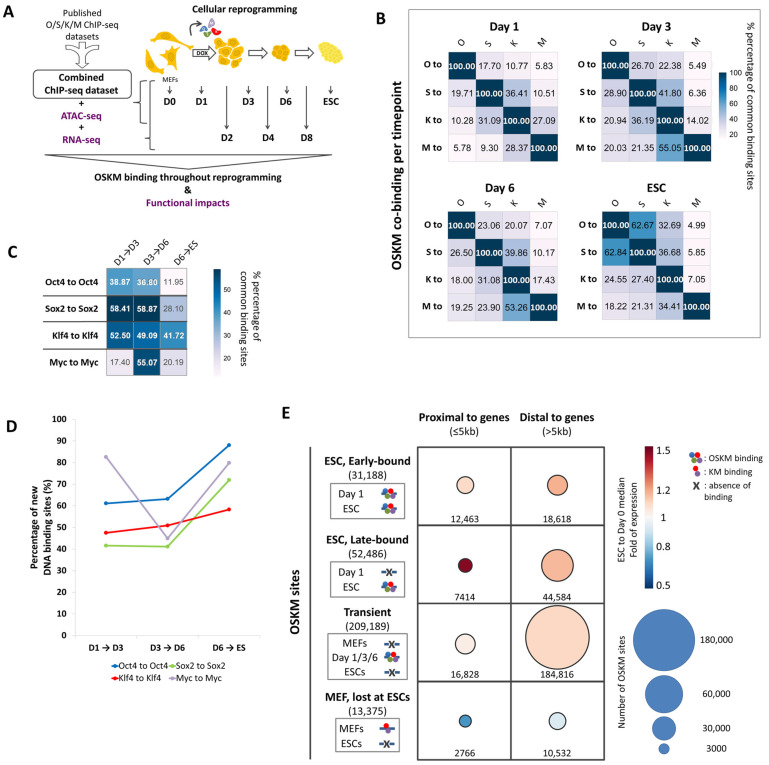
Oct4, Sox2, Klf4 and c-Myc (OSKM) bind to the genome in a dynamic fashion during cellular reprogramming. (**A**) Graphical representation of the experimental approach used to map the OSKM binding sites during cellular reprogramming and their functional output. We integrated published O/S/K/M ChIP-seq experiments to construct a composite ChIP-seq dataset representing the day 0 (D0), day 1 (D1), day 3 (D3), day 6 (D6) time-points together with the Embryonic Stem Cells (ESC) stage. These data were further integrated with results obtained from ATAC-seq at the same time-points and with RNA-seq experiments performed at day 0 (D0), day 1 (D1), day 2 (D2), day 3 (D3), day 4 (D4), day 6 (D6), day 8 (D8) and ESCs. (**B**) Heatmaps depicting the extent of the combinatorial binding of O/S/K/M throughout reprogramming (Day 1, Day 3, Day 6 and ESC). Values are calculated as the percentage of binding sites occupied by the Oct4 (O), Sox2 (S), Klf4 (K) and Myc (M) transcription factors (rows) in combination with each of the other three factors (columns), per time-point. The value 100% depicts the total binding sites occupied by each factor. Compare row name to column name. (**C**) Heatmap depicting the percentage of DNA binding sites occupied by Oct4, Sox2, Klf4 and Myc, which have been preserved between two sequential time-points. For example, the value 38.87% (intersection of Oct4 to Oct4 row with D1 → D3 column) represents the percentage of the Oct4 binding sites at day 1 that have been preserved at day 3. (**D**) Linegraph depicting the mobility of O/S/K/M by plotting the relative number of new binding sites occupied by each of the O/S/K/M between two sequential time-points of reprogramming. (**E**) Schematic representation of the different classes of OSKM binding sites occupied during cellular reprogramming. OSKM are depicted as multi-color circles bound to DNA per time-point (left). The absence of OSKM binding to a specific set of sites at a given time-point as compared to ESCs and vice versa is marked with an “X”. Each class of OSKM sites is divided in regions either proximal (≤5 kb) or distal (>5 kb) relative to the neighboring genes’ TSS. The size of the circles in the table represents the number of OSKM binding sites of each of the corresponding class. The median expression fold between ESCs and Mouse Embryonic Fibroblasts (MEFs, Day 0) for each class of genes is depicted as a heatmap in the figure.

To investigate the complex molecular mechanisms involved in TF-induced cellular reprogramming, we examined the binding of the OSKM factors to the mouse genome by integrating publicly available time-course ChIP-seq datasets [10,16,17,18,37] (Figure 1A, Tables S1 and S2) followed by computational analysis. We anticipated that a large-scale analysis of such integrated data acquired from a number of different experiments using the OSKM MEFs reprogramming system is an important parameter for obtaining a representative picture of the OSKM binding activity. Furthermore, the OSKM ChIP-seq time-course dataset (day 0, day 1, day 3, day 6 of reprogramming and in ESCs) was integrated with ATAC-seq and RNA-seq experiments in order to monitor both the requirements and the functional consequences of OSKM genomic binding. The ESCs RNA-seq data used in our analysis were obtained from our previously published dataset [26] (Table S1).

### 2.2. An Unprecedented Highly Dynamic OSKM Binding During Cellular Reprogramming

Our large-scale analysis revealed that OSK tend to co-bind in a gradually increasing mode from day 1 to day 6 of reprogramming (Figure 1B, Oct4, Sox2 and Klf4 rows), while Myc preferentially co-binds with Klf4 only (Figure 1B, Myc rows) [14,17]. Interestingly, we discovered that the highly preferential Oct4-Sox2 co-binding in ESCs represents a stable state established late during reprogramming, because during the long reprogramming process, Sox2 shares more binding sites with Klf4 than with Oct4 (Figure 1B, compare Sox2 row with Oct4 and Klf4 columns at the different time-points). Furthermore, comparison of the common Oct4 (“Oct4 to Oct4”), Sox2 (“Sox2 to Sox2”), Klf4 (“Klf4 to Klf4”) and Myc (“Myc to Myc”) binding sites between days 1 and 3 and between days 3 and 6 revealed that ~50% of their total binding sites on day 1 are lost on day 3 and, similarly, their binding sites were lost from day 3 to day 6 (Figure 1C, “D1 → D3” and “D3 → D6” columns). Importantly, with the exception of Klf4, OSM, especially Oct4 and Sox2, are highly mobile, preserving only 10–30% of their day 6 sites in ESCs (Figure 1C,D, “D6 → ES” column). These observations strongly suggest that the highly dynamic OSKM binding generates transient chromatin landscapes bearing unique functional potentials via the sequential occupation and abandonment of sites, which altogether, are not similar to the landscape found at the terminal and stable ESC state (Figure 1C,D). Overall, our data suggest that on the road to achieve pluripotency, OSKM are in a constant mobility phase onto chromatin rewiring various enhancer landscapes, thus providing a reasonable molecular explanation for the generation of cellular plasticity in reprogramming [38].

### 2.3. The Combinatorial Binding of OSKM to Early and Late Elements Prefigures the Induction of Pluripotency-Related Genes During Reprogramming

To investigate how the continuously changing OSKM DNA binding landscape during reprogramming culminates in the establishment of the unique stable DNA binding pattern in ESCs, we grouped the total sites occupied by OSKM from the beginning of reprogramming until the acquisition of pluripotency (ESCs) according to their temporal DNA binding pattern. We identified four major classes of sites: the early-bound ESC sites (ESC sites occupied from day 1), late-bound ESC sites (ECS sites occupied after day 3 of reprogramming), transient sites (sites that are occupied transiently during reprogramming), and the MEF sites (sites bound by KM in MEFs and OSKM during reprogramming, but not in ESCs) (Figure 1E). Collectively, during reprogramming, the majority of the total sites (>200,000 sites) bound by OSKM are occupied by these factors transiently only. This finding further underscores the dynamic and the stochastic nature of reprogramming. Importantly, Figure S1 shows that the temporal binding of OSKM to these four classes of sites coincides with epigenetic changes in chromatin. More specifically, the ESC sites (early and late) progressively gain active enhancer histone marks (H3K4me1 and H3K27ac), while they lose the repressive H3K27me3 modification (Figure S1A–C). Accordingly, the transiently-bound OSKM sites temporarily only acquire permissive chromatin marks, with only a small percentage of them maintaining the active mark to ESCs (Figure S1D–F). By contrast, the KM-bound MEF regions progressively lose their active histone marks (Figure S1G–I), a fact that is in agreement with the down-regulation of their associated genes (Figure 1E).

**Figure 2:**
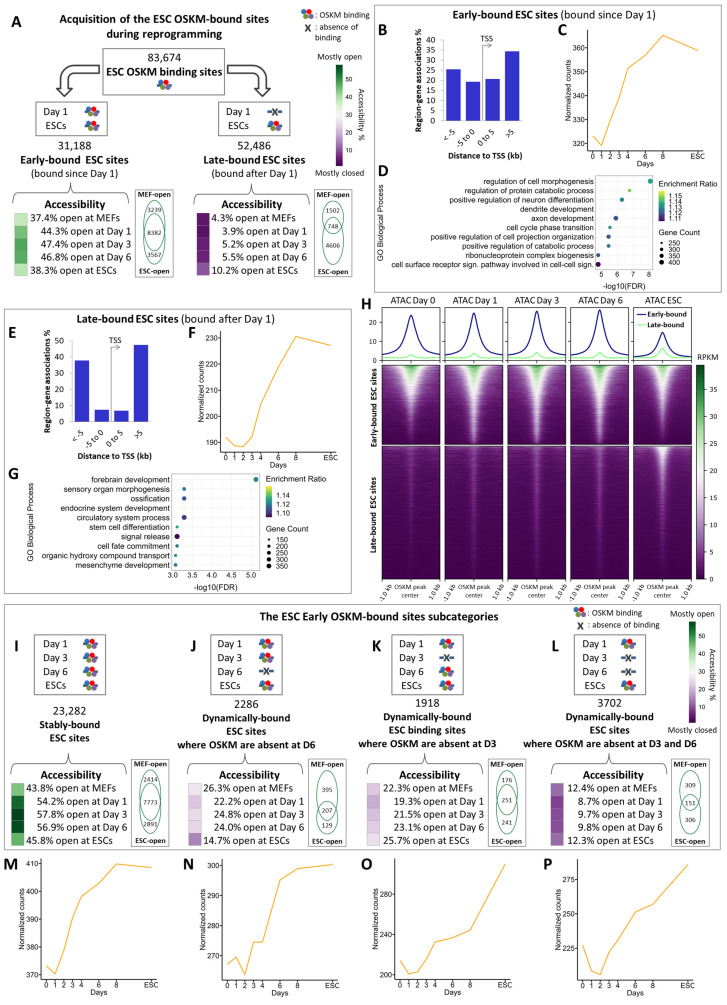
Identification and characterization of the ESC OSKM binding sites acquired during cellular reprogramming. (**A**) Graphical representation summarizing the acquisition of the ESCs OSKM binding sites during reprogramming. OSKM are depicted as multi-color circles bound to DNA. The absence of OSKM binding to a specific set of sites on Day 1 as compared to ESCs is marked with an “X”. The heatmap shown at the bottom part of the figure depicts the percentage of each class of OSKM sites lying in open chromatin regions during reprogramming (MEFs, Day 1, Day 3, Day 6, ESCs). Green and purple colors correspond to the percentage of binding sites accessible per time-point (green for >30%, purple for <30%). The Venn diagrams depict the common chromatin open sites between MEFs and ESCs for each class of sites. (**B**) Association of the early-bound OSKM ESC sites with the neighboring genes using the GREAT algorithm. Depicted is the distribution of the OSKM sites relative to the nearest gene Transcription Start Site (TSS) within 1000kb distance. (**C**) Shown is a line graph depicting normalized counts for the expression of genes associated with the early-bound OSKM ESC sites during cellular reprogramming. The median expression value is shown for each time-point; (**D**) Functional enrichment analysis (over-representation analysis, ORA) of the genes located near the early-bound OSKM ESC sites. Depicted are the top 10 terms as sorted using FDR (FDR < 0.01). The Gene Ontology (GO) Biological Process library (non-redundant terms) was used. (**E**) Same as in B, but for the late-bound OSKM sites. (**F**) Same as in C, but for the late-bound OSKM sites. (**G**) Same as in D, but for the late-bound OSKM sites. (**H**) Summary plots and heatmaps depicting the ATAC-seq signal at the early- and late-bound OSKM ESC sites from -1kb to +1km from the center of the OSKM peaks during cellular reprogramming (Day 0, Day 1, Day 3, Day 6, ESCs). The signal is calculated as RPKM. The OSKM sites are sorted in a descending order based on the ATAC-seq signal in ESCs. (**I**) Graphical representation summarizing the acquisition of the stably-bound OSKM ESC binding sites during reprogramming. OSKM are depicted as multi-color circles bound to DNA. The absence of OSKM binding to a specific set of sites on Day 3, or Day 6, as compared to ESCs is marked with an “X”. The heatmap shown at the bottom part of the figure depicts the percentage of the stably-bound OSKM ESC sites lying in open chromatin regions during reprogramming (MEFs, Day 1, Day 3, Day 6, ESCs). Green and purple colors correspond to the percentage of binding sites accessible per each time-point (green for >30%, purple for <30%). The Venn diagrams depict the common chromatin open sites between MEFs and ESCs. (**J**) As in I, but for the early OSKM ESC sites bound on Day 1, Day 3 and ESCs (Dynamically-bound early ESC sites, where OSKM are absent on Day 6). (**K**) As in I, but for the early OSKM ESC sites bound on Day 1, Day 6 and ESCs (Dynamically-bound Early ESC sites, where OSKM are absent on Day 3). (**L**) As in I, but for the early OSKM ESC sites bound only on Day 1 and ESCs (Dynamically-bound Early ESC sites, where OSKM are absent on Day 3 and 6). (**M**) As in C, but for the stably-bound OSKM ESC sites. (**N**) As in C, but for the early OSKM ESC sites bound on Day 1, Day 3 and ESCs (Dynamically-bound Early ESC sites, where OSKM are absent on Day 6). (**O**) As in C, but for the early OSKM ESC sites bound on Day 1, Day 6 and ESCs (Dynamically-bound Early ESC sites, where OSKM are absent on Day 3). (**P**) As in C, but for the early OSKM ESC sites bound only on Day 1 and ESCs (Dynamically-bound Early ESC sites, where OSKM are absent on Day 3 and 6).

More specifically, we found that of the total 83,674 OSKM binding sites in ESCs (Figure 2A, top panel), 31,188 (37.3%) sites are bound by OSKM from day 1 (early-bound sites) (Figure 2A, left panel), while the remaining 52,486 sites are sequentially occupied at days 3, 6, and later (late-bound sites) (Figure 2A, right panel). Furthermore, we found that the early-bound sites are preferentially located at ±0–5 kb from the Transcription Start Sites (TSSs) of the closest genes (more proximal regions), whereas the late-bound sites are primarily located between 5 and 500 kb from the TSSs (distal regions) (Figure 2B,E). OSKM early binding events coincide with the rapid down-regulation and up-regulation of neighboring genes mainly involved in the somatic state and developmental and housekeeping processes, respectively, whereas the late-bound sites correlate to a more delayed activation (after day 3) of genes involved in developmental and differentiation processes (compare Figure 2C,D with Figure 2F,G).

Intriguingly, we found that the vast majority of the OSKM-bound genes in ESCs (9992 genes) bear both early- and late-bound OSKM sites (Figure S2A, Table S3), thus suggesting that these genes could be regulated in a combinatorial manner by OSKM via widely separated cis-regulatory elements occupied at different times during reprogramming. Some of these genes are induced during reprogramming, such as the critical pluripotent regulators *Pou5f1* (Oct4), *Sox2* and *Nanog* (OSN), and various other reprogramming TFs and cofactors, like *Bhlhe40*, *Ehf*, *Elf3*, *Esrrb*, *Etv5*, *Lin28a/b*, *Nr5a2*, *Sall1/4*, *Tbx3*, *Tfcp2l1*, *Utf1* and *Zic3* (Table S3, Figures S1F and S2C). Importantly, six out of the nine TRs (i.e., *Rcan1*, *Tead4*, *Tfap4*, *Gli2*, *Ovol1* and *Nanog*) previously shown to reconstruct the 9TR gene regulatory network required for cellular reprogramming [10] are also regulated by the combinatorial action of early- and late-OSKM binding at proximal and distal regulatory elements, respectively. Of note, this group of genes includes also TFs that are down-regulated during reprogramming, like *Snai1*, *Twist1/2* and *Zeb1/2*, all of which are involved in maintaining the mesenchyme phenotype [26,39], and thus inhibit reprogramming. Interestingly, although the OSKM ESC early-bound genes display a relatively stable expression pattern during the first 6–8 days of reprogramming, their expression is dramatically decreased towards ESCs, even though OSKM are still bound at their respective DNA elements (Figure S2B), whereas the late-bound OSKM ESC genes are induced at the latest phases of reprogramming (compare Figure S2C and Figure S2D) including, among others, genes involved in extracellular matrix (ECM) organization (Figure S2G and Table S3).

As predicted from our analysis, a significant fraction of the early-bound OSKM sites (37.4%) lie in open chromatin in MEFs (Figure 2A, left panel heatmap, Figure 2H, top panel and Figure S1A–C), bearing also motifs for somatic TFs (e.g., Fosl, Jun and Tead families) (Figure S3A). On the contrary, the OSKM late-bound ESC elements are in a closed chromatin configuration in MEFs, they open in ESCs (Figure 2A, right panel heatmap, Figure 2H, lower panel and Figure S1A–C), contain various motifs for pluripotent TFs, such as Nanog, and are linked to genes involved exclusively in cell fate decision processes (Figure 2G and Figure S3B).

The pioneering activity of OSKM in initiating reprogramming begins with their binding to the early-bound sites, a significant fraction of which is constitutively open in MEFs and remain open throughout reprogramming (23,282 sites), and a fraction of these lie within ESC-specific super-enhancers (SEs) [17,40] (Figure 2I, Table S4). We also note that stable binding of OSKM in these open sites is also marked by the co-presence of somatic TF motifs, suggesting that these regions could play a binary role in both the mesenchymal and the pluripotent state (Figure 2I, Venn diagram, Figure S3C). We assume that OSKM binding to these sites eventually replaces pre-bound somatic TFs (Figure 2M, see a list of genes in Table S3). Intriguingly, a small percentage of early-bound ESC sites close and re-open at various stages of reprogramming (Figure 2J–L), thus causing dynamic changes in the gene expression programs of the associated genes (Figure 2N–P). This finding further underscores the continuously changing chromatin landscape controlled by OSKM.

### 2.4. Identification of Reprogramming-Inducible Enhancers in the Mouse Genome

**Figure 3:**
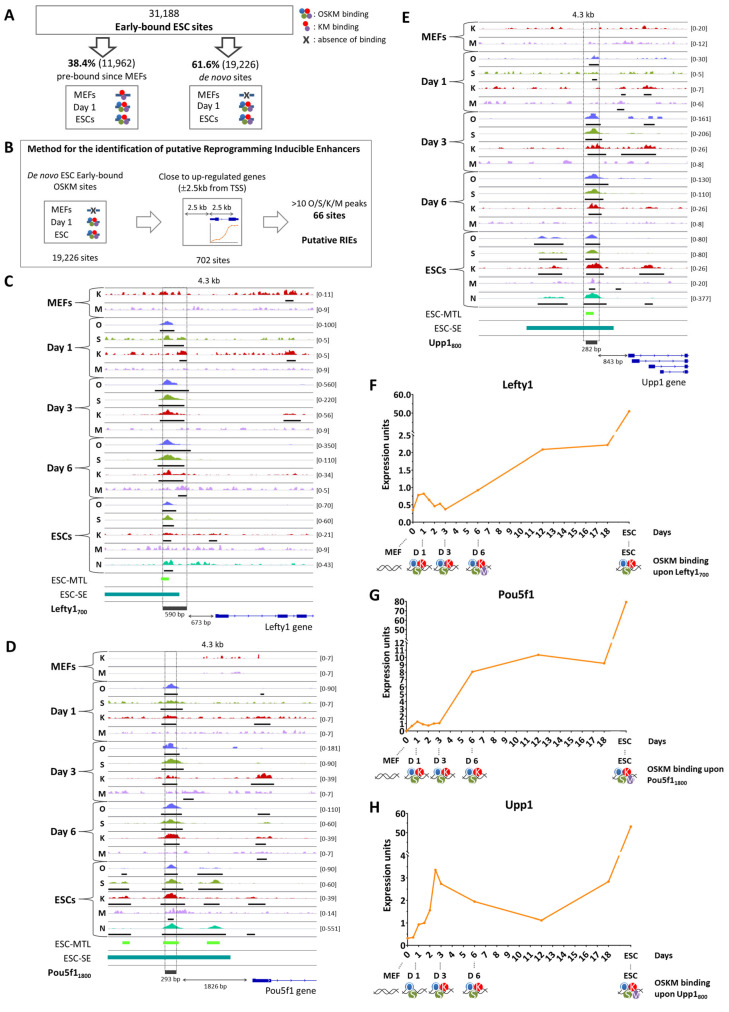
Identification of putative Reprogramming-Inducible Enhancers (RIEs) in the mouse genome. (**A**) Graphical representation summarizing the acquisition of the early-bound OSKM ESC sites. The left panel shows sites that are pre-bound by KM in MEFs, whereas the right panel depicts the de novo sites occupied by OSKM after initiation of reprogramming. OSKM are depicted as multi-color circles bound to DNA. The absence of OSKM binding to a specific set of sites in MEFs as compared to ESCs is marked with an “X”. (**B**) Graphical representation of the unbiased method used to identify putative RIE elements along with the sequential filtering of the OSKM-bound genomic elements. (**C**) Shown are ChIP-seq bigwig files in the Integrated Genome Viewer (IGV) browser depicting the binding of Oct4 (O, blue), Sox2 (S, green), Klf4 (K, red), Myc (M, purple) and Nanog (N, cyan) to Lefty1_700_ putative RIE in MEFs undergoing reprogramming (Day 1, Day 3 and Day 6) and in control MEFs (Day 0) and ESCs. The scale for each snapshot is shown on the right. The binding signal has been calculated as RPKM after subtraction of the input signal from the respective immunoprecipitation (IP) signal. Statistically significant peaks are depicted with a black bar below the respective lane. The ESC Multiple Transcription Factor binding Loci (ESC-MTL) is depicted as a light green bar. The *Lefty1* ESC-specific super-enhancer (ESC-SE) is depicted as a petrol bar. The position of the Lefty1_700_ element is depicted as a dark grey bar at the bottom of the panel. (**D**) As in C, but for the Pou5f1_1800_ element. (**E**) As in C, but for the Upp1_800_ element. (**F**) Shown is a line graph depicting normalized expression units of the *Lefty1* gene during reprogramming. The bottom part of the panel summarizes the binding of OSKM at each time-point (data taken from (**C**)). Oct4 (O) is depicted in blue, Sox2 (S) in green, Klf4 (K) in red and c-Myc (M) in purple. (**G**) As in F, but for the *Pou5f1* gene and the Pou5f1_1800_ element. Data taken from (**D**). (**H**) As in F, but for the *Upp1* gene and the Upp1_800_ element. Data taken from (**E**).

So far, we have provided a comprehensive compilation of putative genomic regulatory regions bound by OSKM during different stages of cellular reprogramming by performing genome-wide occupation analytical studies integrated with gene expression and open chromatin signatures (Figure 1E, Figure 2 and Figure S1). Previous studies have identified a large number of biochemical and functional interactions between O/S/K/M, thus implying that they collaboratively initiate cellular reprogramming [17,22]. This unprecedented and carefully coordinated OSKM dynamic binding along with the binding of other TFs, including the 9TR network, lead to a sequential occupation of markedly different genomic regions between early and later stages of reprogramming, thus driving cells to abandon their mesenchymal phenotype and induce their transition towards pluripotency. For example, only the small percentage of cells in which the 9TR network is successfully reconstructed and activated by the direct action of OSKM will be reprogrammed into iPSCs [10] (Figure S4A–I). For an initial assessment of the existence and the biological relevance of putative Reprogramming-Inducible Enhancers (RIEs), we searched the genome for such enhancers with in vivo reprogramming activity. Furthermore, the identification of RIEs will allow the isolation and study of the precious rare cell populations undergoing successful transition from the somatic to the pluripotent phenotype in real time. Our previous analysis identified early-bound ESC genomic sites (Figure 2A, left panel) implicated in the up-regulation of genes related to the transition to pluripotency (Figure 2C,D and Figure S2C,F). Importantly, 19,226 of these sites are bound by OSKM only after the initiation of reprogramming (Figure 3A, right panel de novo sites), and therefore, we assumed that they could be contained within putative RIEs.

Based on the above, we carefully inspected the ESC genomic sites to assess the value of these elements in functioning as true RIEs. More specifically, we searched the de-novo-acquired OSKM early-bound ESC sites focusing on regions residing up to ±2.5 kb from the TSS of up-regulated genes during reprogramming (Figure 3B). We focused on proximal sites based on previous studies suggesting that it is highly probable for a gene to be regulated by its most proximal enhancer, than from elements dispersed in large distances from the gene [41,42]. Our choice to search for RIEs within the proximal gene regions was a necessary preliminary filtering step in order to reduce the vast number of putative regulatory elements scattered in the genome (>19,000 putative OSKM-bound sites) to a smaller number of DNA elements (702 putative RIEs) located at proximal gene regions. This observation does not exclude the possibility that there exist many additional elements that could also function as RIEs located at distal regions. Importantly, in our survey for RIEs, we did not take into account the biological function of the genes close to these elements, or any data regarding genomic regions previously characterized as regulatory elements. Thus, our RIEs identification approach was based on an unbiased logic. The resulting 702 sites were further filtered to remove loci bearing 10 or less Oct4, Sox2, Klf4 or Myc peaks in total. This filtering step was also important in order to identify DNA elements that are densely and robustly bound by multiple O/S/K/M factors, thus serving as good candidates for functional RIEs working as compact platforms to support direct interactions between the OSKM proteins (i.e., cooperative binding). Based on this assumption regarding the arrangement of O/S/K/M binding sites, we identified 66 co-bound regions of adjacent multiple O/S/K/M peaks, which we hypothesized that could function as putative RIEs (Table S5). Of these, three test enhancers were selected as representative examples for further functional validation analyses: a 600 bp region located ~700 bp upstream of the *Lefty1* gene (“Lefty1_700_” element, Figure 3C), a 300 bp region located ~1800 bp upstream of the *Pou5f1* gene (“Pou5f1_1800_” element, Figure 3D) and a 300 bp region residing ~800 bp upstream of the *Upp1* gene (“Upp1_800_” element, Figure 3E). Our only criteria for choosing these three elements were their property to support high-affinity and dense O/S/K/M binding. *Lefty1* encodes for a protein belonging to the TGF-β family of ligands, which plays a role in determining the left–right symmetry in the developing embryo and it is also a stemness marker [43,44]. *Pou5f1* encodes for the Oct4 transcription factor protein. Oct4 has a vital role in regulation of pluripotency by forming the core of the pluripotency transcription factor network along with Sox2 and Nanog [45]. Finally, *Upp1* encodes for a uridine phosphorylase, an enzyme catalyzing the reversible phosphorolysis of uridine, often found to be up-regulated in rapidly dividing malignant cells [46,47]. All three putative RIEs are stably bound by OSK from day 1 throughout reprogramming to ESCs, with the exception of Upp1_800_, where Klf4 binding occurs from day 3 and afterwards. Importantly, all three elements are bound by Nanog in ESCs (Figure 3C–E). Furthermore, OSK early binding (day 1) correlates with an increase of the corresponding gene expression within the first 24 h (Figure 3F–H). Taken together, these observations support the idea that the above genomic elements could indeed function as RIEs. In addition, ATAC-seq analysis revealed that these sites are occupied by nucleosomes in MEFs, but become accessible early in reprogramming (Figure S5A–C). More specifically, OSK binding at Lefty1_700_ correlates with an immediate opening of the local chromatin, while Pou5f1_1800_ and Upp1_800_ become accessible after day 3. Accordingly, although at the beginning of reprogramming all three putative RIEs are marked by the repressive histone modification H3K27me3, during reprogramming there is a gradual replacement of the repressive mark by the H3K27ac modification, a transcriptionally active promoter/enhancer marker, a finding consistent with their function as RIEs (Figure S5D–F) [17]. Interestingly, all three RIEs coincide with ESC-related Multiple Transcription-factor binding Loci (MTLs)—regions short in size, bound by multiple TFs, which are believed to act as sites of enhanceosome assembly in ESCs (Figure 3C–E, bottom). The elements have been also annotated as ES-specific super-enhancer regions (Figure 3C–E, bottom) [40,48]. Taken together, the Lefty1_700_, Pou5f1_1800_ and Upp1_800_ identified by our unbiased approach (Figure 3B) share many structural characteristics with functional enhancer elements and they are part of previously known regulatory elements in ESCs, and therefore, they could indeed function as true RIEs.

### 2.5. The Upp1_800_ Element Functions as a Reprogramming-Inducible Enhancer Marking Cells Undergoing Reprogramming to Pluripotency

**Figure 4:**
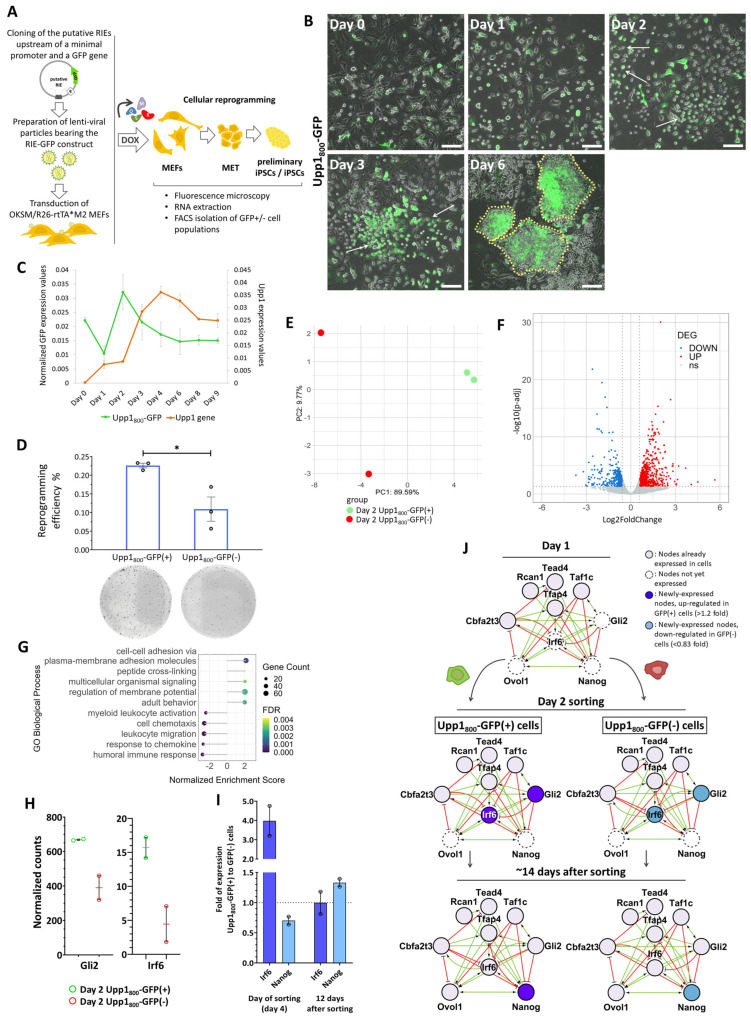
The Upp1_800_ element is a Reprogramming-Inducible Enhancer (RIE) that marks cells undergoing reprogramming and the early induced Pluripotent Stem Cell (iPSC) colonies. (**A**) Shown is the experimental outline used to test the transcriptional capacity of the putative RIEs. (**B**) Representative fluorescence microscopy images taken from a time-course reprogramming experiment using MEFs transduced with the lenti-virus bearing the Upp1_800_-GFP reporter cassette. The brightfield (phase contrast) and fluorescence images were merged in each time-point. The white arrows point to cells abandoning the MEF phenotype (Mesenchymal to Epithelial Transition, MET). Early iPSC formations are indicated with yellow dashed lines. Scale bar: 150 μm. (**C**) Line graph depicting the expression of the endogenous *Upp1* gene (orange line, right axis) in comparison with the expression of the Upp1_800_-GFP transgene (green line, left axis). Shown are the mean expression values from two biological replicates and the standard error. (**D**) Bar graphs showing the iPSCs generation efficiency (%) of the isolated (day 4) Upp1_800_-GFP(+) cells undergoing reprogramming, as compared to the Upp1_800_-GFP(−) cells isolated from the same experiment used as control. Three biological replicates are depicted. The mean and the standard error are also depicted. Unpaired two-tailed t-test was performed (t = 3.525, df = 4, *p*-value = 0.024). Representative images of the Alkaline Phosphatase (AP)-staining plates are also shown at the bottom. *: *p*-value < 0.05. (**E**) PCA analysis of the RNA-seq profile of Upp1_800_-GFP(+) (green) and GFP(−) cells (red) isolated on day 2 of reprogramming. (**F**) Volcano plot depicting the number of DEGs identified between Upp1_800_-GFP(+) and GFP(−) cells. Cut-offs for Volcano plot: *p*-adjusted < 0.05 (horizontal dotted line) and Log_2_FC > 0.58 or <−0.58 (vertical dotted lines). (**G**) Functional enrichment analysis (Gene Set Enrichment Analysis, GSEA) of the genes expressed in Upp1_800_-GFP(+) cells isolated on day 2 as compared to control Upp1_800_-GFP(−) cells. Log_2_FC was used for ranking. The Gene Ontology (GO) Biological Process library (non-redundant terms) was used. Depicted are the top 5 terms with both positive and negative normalized enrichment scores. (**H**) Dot plots depicting normalized counts for the expression of the *Gli2* and *Irf6* genes in the Upp1_800_-GFP(+) and GFP(−) cells, as calculated by RNA-seq on day 2 of reprogramming. Two biological replicates are depicted. The mean and the standard error are also depicted. (**I**) Bar chart depicting the fold change of *Irf6* and *Nanog* expression between the Upp1_800_-GFP(+) and GFP(−) cells on day 4 (day of isolation) and 12 days after isolation of the cells. The dashed line represents the fold of expression equal to 1. Two biological replicates are depicted for each cell type. The mean and the standard error are also indicated. (**J**) Schematic representation of the 9TR-GRN (9 Transcriptional Regulators Gene Regulatory Network) network’s sequential assembly along with the network nodes’ expression status in cells bearing the Upp1_800_-GFP transgene on Day 1 (top panel, before sorting) and after sorting to Upp1_800_-GFP(+) and GFP(−) cells (Day 2 to Day 14 of reprogramming, middle and bottom panel, respectively). The representation is shown according to Papathanasiou et al., 2021 [10]. The green arrows represent the positive effect of one regulator to another. The red lines represent inhibitory effects of one regulator to another.

To investigate whether the three putative regulatory elements act as true RIEs, we generated reporter constructs in which a minimal promoter driving the expression of the *Gfp* gene was fused to the test enhancers immediately upstream, that is, in a position similar to their natural genomic location (Figure 4A, left panel). The resulting constructs were cloned into a vector suitable for the generation of lenti-viral particles. Next, the produced lenti-viruses bearing the putative RIE reporters were used to transduce MEFs with the OKSM transgene incorporated into the *Col1a1* locus and the rtTA*M2 transactivator under the ROSA 26 promoter [49,50]. Interestingly, we found that initiation of cellular reprogramming with the addition of doxycyclin (DOX) is accompanied by the activation of the putative RIEs. We observed a robust *Gfp* expression driven by all three putative RIEs during reprogramming (Figure 4B and Figure S6A,B). More specifically, during the first 3 days of reprogramming, a gradually increasing number of GFP-expressing cells (GFP(+) cells) appeared within the population of cells undergoing reprogramming. Morphological examination revealed that the majority of the GFP(+) cells had abandoned their mesenchymal phenotype as they appeared smaller, round, with an increased number of intercellular contacts (especially since day 3) (arrows in Figure 4B and Figure S6A,B). This is a profound alteration characterizing cells that undergo MET [8]. Importantly, many of the GFP(+) cells cluster within the early iPSC colonies, especially those cells expressing the Lefty1_700_ and Upp1_800_ reporters (see day 6 in Figure 4B and Figure S6A). In contrast, expression of the Pou5f1_1800_ reporter appears to be less restricted to the early iPSC colonies. These experiments strongly suggest that Lefty1_700_, Pou5f1_1800_ and Upp1_800_ function as RIEs and they mark cells undergoing phenotypical transitions during reprogramming. Significantly, the Lefty1_700_ and Upp1_800_ reporters not only specifically mark activation of *Gfp* gene expression after initiation of reprogramming, but they also mark the cells that will form the early iPSCs colonies (Figure 4B and Figure S6A). On the other hand, the Pou5f1_1800_ RIE suffices to warrant transcriptional activation in the context of reprogramming only, without marking specifically the early iPSCs formations (Figure S6B).

Next, we asked whether these putative RIEs suffice to regulate the expression of their adjacent genes during reprogramming. Thus, we compared the expression pattern of the endogenous genes (*Lefty1*, *Pou5f1* and *Upp1*) with the expression of the exogenous *Gfp* controlled by the respective regulatory elements (Figure 4C and Figure S6C,D). Remarkably, we found that both the Lefty1_700_ and Upp1_800_ GFP reporters display expression patterns that are similar to their endogenous counterparts, thus implying that these elements are the main regulatory enhancers controlling the expression of these genes. Of note, the small temporal difference (1–2 days) observed between the *Gfp* and the endogenous gene expression pattern could be explained by the fact that as the exogenous RIE–GFP construct can be incorporated in various random open chromatin genomic sites, it may lack an appropriate epigenetic landscape, which normally could delay the endogenous gene expression. Since the Lefty1_700_ element simulates the expression pattern of the endogenous gene during the first 6 days only, followed by a gradual decrease in its activity, it implies that this element is not sufficient to support *Lefty1* gene expression throughout the entire course of reprogramming, but it suffices only for the initial stages of the process (Figure S6C). On the other hand, the Pou5f1_1800_ element did not mimic the expression program of the endogenous gene (Figure S6D), thus suggesting that *Pou5f1* expression is controlled by additional regulatory elements during reprogramming. 

### 2.6. The Cells “Marked” by the Reprogramming-Inducible Enhancer Upp1_800_ Element Achieve Earlier and More Robust Induction of the 9TR Network Leading to Efficient Reprogramming

We described above the integration of multiple features that led us to conclude that the Upp1_800_ and Lefty1_700_ enhancers function as autonomous RIEs activated early during reprogramming, marking the cells undergoing MET in early iPSC colonies (Figure 4B and Figure S6A). Therefore, we used the RIE Upp1_800_-GFP reporter as a tool to isolate the cells undergoing reprogramming by monitoring GFP expression in order to explore their transcriptional and phenotypical properties (Figure 4A, right panel). We chose the Upp1_800_-GFP reporter because it accurately recapitulates the expression of the endogenous *Upp1* gene throughout reprogramming (Figure 4C). FACS-isolated Upp1_800_-GFP(+)and GFP(−) cells on day 4 of reprogramming were re-plated in parallel and their reprogramming efficiency was calculated. We found that the GFP(+) cells produced two times more AP-positive iPSC colonies as compared to the GFP(−) cells (Figure 4D). This finding is in agreement with the reporter assays, where we observed high-level expression of Upp1_800_ driven GFP expression in early iPSC colonies (Figure 4B and Figure S6E).

To investigate the molecular signature of the Upp1_800_-GFP(+) cells that enables them for efficient reprogramming, as opposed to the GFP(−) cells, we performed RNA-seq experiments side by side with the GFP(−) cells used as a control to “capture” the underlying molecular pathways supporting highly efficient reprogramming. Comparison of the RNA-seq data between GFP(+) and GFP(−) cells revealed that these two subpopulations are characterized by distinct transcriptome profiles, since we identified a significant number of up- and down-regulated Differentially Expressed Genes (DEGs) (Figure 4E,F). More specifically, the GFP(+) cells, where the Upp1_800_ RIE is activated, bear 718 up-regulated and 394 down-regulated DEGs as compared to the GFP(−) cells (Figure 4F, Table S6). Functional enrichment analysis (Gene Set Enrichment Analysis, GSEA) revealed that the up-regulated genes are related to intercellular adhesion and various signaling processes, whereas down-regulated genes are involved in chemotaxis and response to chemokines, processes indicative of the complex cell–cell interactions and the phenotypic alterations occurring during the early days of reprogramming (Figure 4G).

To further investigate the reconstruction of molecular networks marking the reprogrammable cells, we used the MCODE algorithm [51] to cluster groups of DEGs involved in the various biological processes described above (Figure S7). This detailed analysis verified that, indeed, in the GFP(+) cells, clusters of down-regulated genes are formed that are involved in chemotaxis, in responses to extracellular signals (Figure S7, Clusters 1–2), in cell migration and EMT (Figure S7, Clusters 1–2, 4–5, 9). For example, *Bmp4*, an Epithelial to Mesenchymal Transition (EMT)-promoting gene functioning as a reprogramming inhibitor [52,53], is down-regulated in GFP(+) cells, thus relieving the cells from a reprogramming barrier (Figure S7, Cluster 5). On the contrary, genes involved in the establishment of cell adhesion and epithelial functions are up-regulated, including, among others, *Dcn* and various basement membrane components like laminin and collagen IV genes (Figure S7, Clusters 5, 9). These genes function by facilitating MET, a requirement for reprogramming. Furthermore, genes involved in embryonic development and morphogenesis are also up-regulated in Upp1_800_-GFP(+) cells (Figure S7, Clusters 5, 7–8). Of these, an important example includes the telomerase reverse transcriptase gene (*Tert*) (Figure S7, Cluster 5). This finding further validates our experimental approach, since regulation of telomere length by the induced *Tert* is critical for the achievement of the high pluripotency potential of ES and iPS cells [54,55,56,57]. In this context, we also identified that *Tpp1*, a gene regulating Tert binding to telomeres during MEF reprogramming [58], is also up-regulated in the Upp1_800_-GFP(+) cells (Table S6). As expected, we showed that a number of Hox TFs, previously implicated in high-efficiency reprogramming [59], are also up-regulated in the RIE-GFP(+) cells (Figure S7, Cluster 7). Taken together, these data further validate our previous results, indicating that the RIEs could mark specific cells in a population that undergo global morphological and molecular alterations in the beginning of reprogramming. More specifically, we have been able to “tag” cells that undergo MET in which specific sets of genes involved in the transition have been activated.

The conclusion from these data was further supported from single-cell RNA-seq experiments performed during reprogramming. We reanalyzed the single-cell RNA-seq data of Schiebinger et al. [60] by monitoring the expression of *Upp1* and found that it is expressed at very low levels and in a small percentage of cells on day 2, but at later time-points (day 4 and afterwards) its expression is gradually increased and becomes uniform (in every cell) especially in iPS cells (Figure S8A,B). Importantly, *Upp1* expression occurs in the same iPS cells in which the pluripotency marker *Utf1* is expressed, thus strengthening our conclusion that *Upp1* prefigures and marks the pluripotency state. In contrast, and in support of our main conclusion, we discovered a strong expression anticorrelation between *Upp1* and the mesenchymal marker *Tagln* (Figure S8C,D). Taken together, these data strongly support our conclusions that the Upp1_800_ enhancer can be used as a marker to study the trajectories towards pluripotency. 

We have previously shown that the small percentage of cells completing reprogramming reconstruct a dynamic gene regulatory network known as 9TR-GRN [10]. We found that the TFs *Gli2* and *Irf6*, which are direct targets of OSKM and components of the 9TR-GRN, are up-regulated in the Upp1_800_-GFP(+) cells (Figure 4H, Figures S6F and S7I). Next, we sorted Upp1_800_-GFP(+) cells and GFP(−) cells on day 4 of reprogramming followed by replating to evaluate their molecular characteristics at later time-points. First, we examined the EMT/mesenchymal markers *Snail1* and Vimentin (*Vim*) along with the 9TR-GRN members *Irf6* and *Nanog*, 12 days after cell sorting and re-plating (Figure 4I and Figure S6G). We found that the GFP(+) cells bear decreased levels of the EMT master regulator *Snail1*, despite the fact that *Snail1* expression was nearly equal between the two cell populations on day 4 before re-plating (Figure S6G, left panel). This observation suggests that the GFP(+) cells, which over-express *Gli2* and *Irf6* early after OSKM induction, abandon the mesenchymal phenotype more efficiently. Interestingly, the classic EMT marker *Vim* is down-regulated in the GFP(+) cells as early as from day 4 (when the sorting is performed) (Figure S6G, right panel), in agreement with our previous data indicating that the Upp1_800_-GFP(+) cells have epithelial characteristics (Figure 4B,G and Figure S7E,I). Intriguingly, *Irf6* is expressed at four times higher levels in Upp1_800_-GFP(+) than in GFP(−) cells on day 4, but later these expression levels are similar (Figure 4I). This implies that the RIE Upp1_800_ marks cells acquiring a temporal “advantage” over the rest of the population in reconstructing the 9TR-GRN network. As a result, these cells could acquire earlier and more efficiently various pluripotent molecular components. Consistent with this is our observation that 12 days after sorting, the GFP(+) cells express more *Nanog* transcripts than the GFP(−) cells (Figure 4I). Taken together, the data described above strongly suggest that the RIE Upp1_800_ is an efficient enhancer-trap for the early identification and isolation of cells entering the reprogramming trajectory.

## 3. Discussion

Cellular reprogramming is a stepwise stochastic process characterized by the emergence of multiple intermediate cell states towards MET from which a small percentage of cells continues to be reprogrammed to achieve pluripotency [3,4,5,10]. Reactivation of the pluripotency gene regulatory network by OSKM requires the rewiring of many gene regulatory elements to permanently inactivate somatic enhancers, to transiently activate enhancers of genes required for initiation of the process, and finally to activate the pluripotency enhancers. In this study, we have highlighted the complexity of OSKM action by creating a composite OSKM ChIP-seq dataset [10,16,17,18,37] used to map in detail the association of the reprogramming factors with the genome to reveal how they orchestrate the gradual acquisition of pluripotency. Contrary to many previous studies, we focused on the temporal and permanent OSKM binding patterns to different genomic elements, aiming to examine how the OSKM ESC binding pattern is achieved and established during reprogramming. Within this context, we identified novel putative regulatory elements bound by OSKM in a complex and dynamic fashion, some of which are capable of functioning as RIEs. We have exploited the transcriptional regulatory property of these elements and isolated cells undergoing reprogramming in real time. Their characterization not only independently validated our previously identified 9TR-GRN as a critical player of reprogramming, but importantly, it also revealed the simultaneous reconstruction of additional reprogramming-specific gene networks controlling the global morphological and molecular alterations required for achieving pluripotency.

We discovered that of the 83,674 genomic sites occupied by OSKM in ESCs, 11,962 sites are pre-bound in MEFs by the endogenously expressed KM, and then they are immediately co-occupied, at least some of them, by OS from day 1 (Early ESC sites). These results suggest that pre-bound KM recruit OS via cooperative interactions. This is an important observation because the role of Myc is usually overlooked in reprogramming and it is often omitted from the transcription-factor “cocktails” [61,62]. However, even in the absence of Myc co-expression with OSK, it is still present in MEFs and its inhibition has dramatic effects in the reprogramming efficiency [21,61].

In contrast to the stably bound OSKM throughout reprogramming, a large number of genomic sites are occupied by OSKM (individually or as a group) in a temporal manner. Apparently, during reprogramming, OSKM become highly mobile by constantly binding to new sites and/or abandoning others inducing alterations in chromatin structure and the expression of the nearby genes. Thus, the dynamic binding of OSKM is directly related to the re-organization of chromatin and the gradual establishment of the pluripotent epigenetic landscape. It is conceivable to assume that, at least in part, the inherent stochasticity of cellular reprogramming is due to the low percentage of cells that will be able to establish the correct pattern of dynamic OSKM binding suitable for orchestrating the correct transcriptional programs towards pluripotency.

An interesting implication of the dynamic OSKM binding patterns described herein stems from the fact that Klf4 appears to regulate the formation of “hubs” composed of pluripotency genes and ESC super-enhancers [37]. Thus, we propose that the temporal pattern of Klf4 binding during reprogramming shown here may regulate the instruction of different hubs consisting of different enhancers associated transiently with the same or different genes during reprogramming. Based on the above, we propose that single genes may “use” multiple OSKM-bound enhancer elements, loaded at different time-points, to ensure the execution of an accurate reprogramming expression pattern.

Our extensive characterization of the various classes of OSKM-bound elements and their association with pluripotency genes prompted us to test whether some of these elements can be used as enhancer-traps to isolate and study the small percentage of cells that are stochastically “chosen” to be reprogrammed. We developed and applied an unbiased method based on the OSKM global binding pattern for the identification of putative RIEs among the early-bound regions. We identified 66 putative RIEs, including the Lefty1_700_, Pou5f1_1800_ and Upp1_800_ elements. We showed that all three elements function as true reprogramming-inducible enhancers. Interestingly, these elements map within ESC-specific super-enhancers [40] and Multiple Transcription Factor binding loci (MTLs) [48]. In addition, the Lefty1_700_, Pou5f1_1800_ and Upp1_800_ elements have been previously implicated in regulation of expression of their associated genes, thus validating our unbiased approach used to identify RIEs. More specifically, Lefty1_700_ is localized within the well-studied proximal *Lefty1* enhancer, which is bound by Oct4 and Sox2 and is responsible for *Lefty1* expression in the early mouse embryo and in ESCs [63,64,65]. On the other hand, Upp1_800_ overlaps with a 1.2 kb regulatory region capable of inducing reporter gene expression in various cells lines [66]. Finally, Pou5f1_1800_ resides within the distal Oct4 enhancer required for its expression in ESCs and in the pre-implantation blastocysts [67,68,69,70,71].

We found that, at least one of these RIEs, the Upp1_800_ element, when fused to a minimal promoter-*Gfp* cassette, can accurately reproduce the expression of the endogenous gene specifically in cells undergoing reprogramming. Therefore, we used the Upp1_800_-GFP reporter to isolate GFP(+) and GFP(−) cells during the first days of reprogramming. Importantly, the GFP(+) isolated cells have twice as much the capacity to complete their reprogramming process as compared to the control GFP(−) cells, since we showed that they acquire more efficiently the epithelial phenotype. This observation is also supported by our finding that genes involved in MET/EMT are differentially expressed in the Upp1_800_-GFP(+) cells (e.g., *Dcn*, *Col4a1/2*, *Lama2*, *Pdgfrb*, *Serpine1/2*, *Slit3*, *Tagln*, *Thbs2*, etc.). Importantly, we found that the GFP(+) cells express at high levels the 9TR-GRN members *Gli2* and *Irf6*, as early as on day 2 of reprogramming, leading to a more robust activation of the 9TR network (Figure 4J). As *Nanog* induction is the final component required for reconstructing the 9TR-GRN network, its efficient up-regulation paves the route to pluripotency.

Furthermore, it has been shown that cells capable of experiencing efficient reprogramming trajectories exhibit a more extensive binding of Oct4 and Sox2 to chromatin, and especially to promoter proximal regions [18]. In agreement with this, we found that Upp1_800_ is densely and robustly bound by OSKM. In summary, by using the OSKM complex DNA binding patterns during reprogramming together with gene expression data as guides, we were able to accurately identify functional enhancers induced in reprogramming (RIEs). We took advantage of the ability of such elements to be efficiently “read” by OSKM only in the small percentage of cells undergoing reprogramming and have developed a tool that, in contrast to previous methods that are based on the detection of protein markers [13,20,31,32,72,73,74], is capable of detecting and isolating the precious population of cells undergoing reprogramming in real time. We believe that our results may have important implications in the development of methods to achieve high reprogramming efficiencies for medical applications.

## 4. Materials and Methods

### 4.1. Experimental Protocols

#### 4.1.1. MEFs Isolation from Mouse Embryos

For our cellular reprogramming experiments, we used mouse embryonic fibroblasts (MEFs) from cross-bred homozygous transgenic mice originating from the B6;129S4-Col1a1^tm1(tetO-Pou5f1,-Klf4,-Sox2,-Myc)Hoch^/J (RRID:IMSR_JAX:011001) and B6.Cg-Gt(ROSA)26Sor^tm1(rtTA*M2)Jae^/J (RRID:IMSR_JAX:006965) strains (“4F-MEFs”) [49,50]. MEFs were isolated as described before [10]. In brief, mouse embryos E13.5-14.5 were harvested from euthanized pregnant female mice. After removing the head, the heart and the liver, the embryonic tissue was chopped up using a razor blade and turned into single-cell suspension by incubation in 0.25% Trypsin solution in PBS (Gibco™, Thermo Fisher Scientific, Waltham, MA, USA, #15090046) and multiple passes through 18G and 21G syringes. MEFs were cultured at 37 °C and 5% CO_2_ concentration, in high-glucose DMEM (Sigma-Aldrich, Merck SA, Athens, Greece, D6429) supplemented with heat-inactivated FBS at 10% final concentration (Pan Biotech, Aidenbach, Germany, P30-1985), 1x GlutaMAX™ Supplement (Gibco™, Thermo Fisher Scientific, Waltham, MA, USA, #35050-061), 1x MEM Non-Essential Amino Acids Solution (Gibco™, Thermo Fisher Scientific, Waltham, MA, USA, #11140-050) and Penicillin-Streptomycin mix diluted at 1/100 (Gibco™, Thermo Fisher Scientific, Waltham, MA, USA, #15140-122).

#### 4.1.2. Cellular Reprogramming Protocol

An appropriate number of “4F-MEFs” (depending on the protocol) was placed in plates (day 0), and cellular reprogramming (OKSM over-expression) was induced with the addition of doxycycline (DOX) at 2 ug/mL (Sigma-Aldrich, Merck SA, Athens, Greece, D9891) in MEF medium as described above, but supplemented with 15% FBS. The medium was refreshed every two days. Upon the emergence of the first early iPSC colonies (around day 8), the medium was replaced with ESC medium: KnockOut™ DMEM (Gibco, Thermo Fisher Scientific, Waltham, MA, USA, #10829018), 20% Pansera FBS designed for ESCs (Pan Biotech, Aidenbach, Germany, P30-2602), 1x GlutaMAX™ Supplement (Gibco™, Thermo Fisher Scientific, Waltham, MA, USA, #35050-061), 1x MEM Non-Essential Amino Acids Solution (Gibco™, Thermo Fisher Scientific, Waltham, MA, USA, #11140-050), Penicillin-Streptomycin mix diluted at 1/100 (Gibco™, Thermo Fisher Scientific, Waltham, MA, USA, #15140-122) and 20 ng/mL mLIF (Santa Cruz Biotechnology, Dallas, Texas, USA, sc-4378).

#### 4.1.3. Nanog Chromatin Immunoprecipitation Followed by High-Throughput Sequencing

ChIP was performed as described before [10]. Cells were cross-linked with 1% formaldehyde at room temperature for 10 min, and then quenched with 0.125 M glycine for 5 min. Cells were washed with PBS and harvested with scraper. Lysis was performed in Lysis buffer [50 mM Hepes (pH 7.9), 140 mM NaCl, 1mM EDTA, 10% glycerol, 0.5% NP-40, 0.25% Triton X-100], washed in Wash buffer [10 mM Tris-HCL (pH 8.1), 200 mM NaCl, 1 mM EDTA (pH 8.0), 0.5 mM EGTA (pH 8.0)] and then resuspended in Sonication buffer [0.1% SDS, 1 mM EDTA, 10 mM Tris (pH 8.1)]. Chromatin was sheared to 200–500 bp fragments in the Covaris S2 sonicator using TC12 × 12 mm tubes (Tube AFA Fiber and Cap, Covaris, Woburn, MA, USA) for 12 min (200 cycles per burst, 75 duty factor, 25 peak power). After the sonication, we added Triton X-100 and NaCl to reach a final concentration of 1% and 150 mM, respectively. The chromatin was then centrifuged and filtered via a 0.2 um filter syringe. An amount of 10 ug of chromatin was incubated with 3,6ug rabbit IgG (crude serum) and 10 ug anti-Nanog (Cell Signaling Technology, Danvers, MA, USA, Cat# 8785, RRID:AB_11220438) at 4 °C overnight. The next day, pre-equilibrated protein G DynabeadsTM (Thermo Fisher Scientific, Waltham, MA, USA, 10004D) in IP buffer [0.1% SDS, 1 mM EDTA, 10 mM Tris (pH 8.1), 1% Triton X-100, 150 mM NaCl] were mixed with the chromatin and antibody solutions. The mix was left to incubate for 1 h at room temperature. Next, the beads were washed with Low salt [0.1% SDS, 1% Triton X-100, 2 mM EDTA, 20 mM Hepes-KOH (pH 7.9), 150 mM NaCl], High salt [0.1% SDS, 1% Triton X-100, 2 mM EDTA, 20 mM Hepes-KOH (pH 7.9), 500mM NaCl] and LiCl buffer [100mM Tris-HCl (pH 7.5), 0.5 M LiCl, 1% NP-40, 1% Sodium deoxycholate]. The immunoprecipitated chromatin was digested with Proteinase K (Roche Life Science, Marousi, Greece, 03115828001) at 50 °C for 15 min, followed by incubation with RNase A at 65 °C overnight and an extra step of Proteinase K for 1 h at 50 °C. The released DNA was cleaned up using a NucleoMag clean-up and selection kit (Macherey-Nagel, Dueren, Germany, 744970) and the elution was performed in TE/10 buffer. Eluted DNA was then used for downstream analyses.

The Nanog ChIP-seq library construction and sequencing were carried out at the Greek Genome Center (GGC) of BRFAA. Libraries were generated with NEBNext^®^ Ultra II™ DNA Library prep Kit for Illumina^®^ (NEB, Ipswich, MA, USA, E7645L), as indicated by the manufacturer’s guidelines using 10 ng of DNA (chromatin that was not incubated with the antibody was used as input DNA). The quality of the generated libraries was validated with the Agilent Bioanalyzer DNA 1000 chip (Agilent, Santa Clara, CA, USA). Subsequently, the libraries were quantitated with the Qubit™ High Sensitivity (HS) spectrophotometric method (Invitrogen, Thermo Fisher Scientific, Waltham, MA, USA) and pooled in equimolar amounts for sequencing. Approximately, 25 million, 101 bp-long, single-end reads were generated with Illumina NovaSeq 6000 sequencer (Illumina, San Diego, CA, USA). The data of the Nanog ChIP-seq experiment are deposited in the Gene Expression Omnibus data repository under the GEO accession ID GSE274131.

#### 4.1.4. ATAC-Seq

ATAC-seq libraries from different time-points were prepared starting from 50,000 cells, following previously published protocols [75,76,77,78]. Briefly, cell pellets were lysed in Lysis Buffer [10 mM Tris-HCl, pH 7.5, 10 mM NaCl, 3 mM MgCl_2_, 0.1% NP-40, 0.1% Tween-20, 0.01% Digitonin (Promega, Madison, WI, USA, G9441)] for 3 min on ice, washed with Wash Buffer [10 mM Tris-HCl, pH 7.5, 10 mM NaCl, 3 mM MgCl_2_, 0.1% Tween-20] and centrifuged at 500 g for 10 min at 4 °C. Each pellet (nuclei) was resuspended in 50ul Transposition reaction mix [1x Tagment DNA Buffer (Illumina, San Diego, CA, USA, 15027866), 16.5 uL 1x PBS, 0.1% Tween-20, 0.01% Digitonin (Promega, Madison, WI, USA, G9441), 2.5 uL Tn5 Transposase (Tagment DNA Enzyme 1, Illumina, San Diego, CA, USA, 15027865)] and incubated at 37 °C for 30 min on a thermomixer at 1000rpm. DNA purification was performed using the Qiagen MinElute Reaction Cleanup Kit (Qiagen, Venlo, The Netherlands, 28204). Library purification was performed with the AMPure XP beads (Beckman Coulter, Indianapolis, IN, USA, A63880). The quality of libraries was assessed using an Agilent 2100 Bioanalyzer (Agilent, Santa Clara, CA, USA) and libraries were quantified with the Qubit™ High Sensitivity (HS) spectrophotometric method (Invitrogen, Thermo Fisher Scientific, Waltham, MA, USA). Libraries were sequenced paired-end using a NovaSeq 6000 sequencer at the Greek Genome Center at BRFAA. The data of the ATAC-seq experiment are deposited in the Gene Expression Omnibus data repository under the GEO accession ID GSE274130.

#### 4.1.5. Cloning of Regulatory Elements and Construction of Enhancer Reporters

We isolated the three identified enhancer elements (Lefty1_700_, Pou5f1_1800_, Upp1_800_) from the mouse genome via PCR using specific primer pairs (Table S7). The primers were designed to enclose the identified OSKM peaks of the respective RIE element in the smallest amplicon size possible. For the construction of our enhancer reporters, we utilized the plasmidic vector LeGO-G2 [79]. LeGO-G2 was a gift from Boris Fehse (Addgene plasmid #25917; http://n2t.net/addgene:25917 (accessed on 3 December 2024); RRID: Addgene_25917). The strong regulatory element SFFV upstream of the *Egfp* gene was removed and replaced each time by one of the three isolated enhancer elements and a minimal promoter sequence. As minimal promoter was used either the endogenous proximal promoter sequences of each of the three genes (Table S7), or the SCP1 element [80], which was isolated from the pSTARR-seq_human vector [81]. pSTARR-seq_human was a gift from Alexander Stark (Addgene plasmid #71509; http://n2t.net/addgene:71509 (accessed on 3 December 2024); RRID: Addgene_71509). The two types of promoters (SCP1 and endogenous) did not affect the pattern of *Egfp* expression and, thus, were used interchangeably in our reporter assays. LeGO-G2 was used as a positive control in our experiments since it showed stable *Egfp* expression over time.

#### 4.1.6. Generation of Lenti-Viral Particles

To introduce our constructs into the MEF genome, we produced lenti-viral particles via transfection in HEK293T cells (Human Embryonic Kidney 293T; RRID:CVCL_0063). The HEK293T cells were transfected with a combination of the plasmids pMD2.G, psPAX2 and each one of our constructs (separately) using the classic calcium phosphate method. The day after the transfection, the supernatant was replaced with fresh medium and 3 days after the medium change, the lenti-viral particles were harvested. HEK293T were cultured at 37 °C and 5% CO_2_ concentration, in high-glucose DMEM (Sigma-Aldrich, Merck SA, Athens, Greece, D6429) supplemented with heat-inactivated FBS at 10% final concentration (Pan Biotech, Aidenbach, Germany, P30-1985), 1x GlutaMAX™ Supplement (Gibco™, Thermo Fisher Scientific, Waltham, MA, USA, #35050-061), 1x MEM Non-Essential Amino Acids Solution (Gibco™, Thermo Fisher Scientific, Waltham, MA, USA, #11140-050) and Penicillin–Streptomycin mix diluted at 1/100 (Gibco™, Thermo Fisher Scientific, Waltham, MA, USA, #15140-122). Plasmids pMD2.G (Addgene plasmid #12259; http://n2t.net/addgene:12259 (accessed on 3 December 2024); RRID:Addgene_12259) and psPAX2 (Addgene plasmid #12260; http://n2t.net/addgene:12260 (accessed on 3 December 2024); RRID:Addgene_12260) were a gift from Didier Trono.

#### 4.1.7. Reporter Assays

- MEFs transduction and cellular reprogramming

For the reporter assays, we transduced ~0.8–1 × 10^6^ early-passage “4F-MEFs” with the above described lenti-viral particles in the presence of polybrene at a final concentration of 6 ug/mL (Sigma-Aldrich, Merck SA, Athens, Greece, H9268) overnight, followed by change of medium. After 2–3 days, an appropriate number of transduced-“4F-MEFs” (depending on the assay) was re-plated and cellular reprogramming was induced with the addition of DOX (day 0), as described above.

- Microscopy

A total of ~600,000 “4F-MEFs” transduced with the enhancer reporters were plated in 10 cm plates and cellular reprogramming was induced after 24 h by the addition of DOX, as described above. Cells were observed regularly under the microscope for the monitoring of the reprogramming process and the detection of GFP(+) cells. A Leica DM IRE2 inverted microscope was used (phase contrast and fluorescence microscopy) with an ORCA-Flash 4.0 LT digital camera (Hamamatsu, C11440-42U, SN 001432). The images were captured with the HCImage Live software (Hamamatsu, v4.3.1.3) and processed using FIJI/ImageJ (1.54f) [82]. Brightness adjustments were made uniformly to all the images of a single experiment in order to correctly depict the increase/decrease of GFP intensity between the different time-points of each experiment. The pixel intensities of the images in Figure S6E were transformed using the LOG function to be able to depict both time-points with very low and very high GFP intensity.

- Reporter assay for the comparison of exogenous-GFP and endogenous gene expression pattern (Figure 4C and Figure S6C,D)

A total of ~70,000 “4F-MEFs” transduced with the enhancer reporters were plated in 6-well plates and cellular reprogramming was induced the next day by the addition of DOX, as already described. For each enhancer reporter, two biological replicates were prepared. Cells were collected using 1x Trypsin solution (Gibco™, Thermo Fisher Scientific, Waltham, MA, USA, #15090046) at different time-points for RNA extraction.

- Sorting of Upp1_800_-GFP(+) and GFP(-) cells for transcriptome analysis and reprogramming efficiency calculation

A total of 1.5–3 × 10^6^ transduced “4F-MEFs” were plated in 10 cm plates and reprogramming was initiated after 24 h (day 0). At least two biological replicates were prepared for each experiment. At day 2 (for day 2 Upp1_800_ RNA-seq) or day 4 (for efficiency calculation of Figure 4D and transcriptome analysis of Figure 4I and Figure S6G), cells were harvested using 1× Trypsin solution (Gibco™, Thermo Fisher Scientific, Waltham, MA, USA, #15090046), washed with PBS and stained with 0.3 uM DAPI for 5–10 min (Roche Life Science, Marousi, Greece, 236276). Next, cells were washed again with PBS and resuspended in cold Sorting buffer [1x PBS, 5% FBS, 1 mM EDTA] to a final concentration of 5–10 × 10^6^ cells/mL. Cell aggregates were removed prior to sorting with 50 um strainer cap filters (BD Biosciences, San Jose, CA, USA, 340629). GFP(+) and GFP(−) sorted cells aimed for day 2/day 4 transcriptome analyses (RNA-seq and analyses of Figure 4I and Figure S6G, respectively) were used directly for RNA extraction (100–200,000 cells per sample). For reprogramming efficiency calculation, alive GFP(+) and GFP(−) cells were counted using Trypan blue and plated in equal numbers on 12-well plates (70–80,000 cells per well). After letting cells overcome stress and expand for almost 10–12 days, they formed healthy iPSC colonies. To compensate for inequalities in cell duplication rates caused by the stress of the sorting process, before estimating the reprogramming efficiency, we re-plated equal numbers of GFP(+) and GFP(−) cells in 6-well plates (100–200,000 cells per well) covered with gelatin (Sigma-Aldrich, Merck SA, Athens, Greece, G1393) and feeders (C57Bl/6 MEFs treated with 10ug/mL Mitomycin C for 2.5–3 h). iPSC colonies were left to grow and were cultured 1 week without DOX, prior to AP staining. GFP(+) and GFP(−) sorted cells aimed to be used for transcriptome analysis at a later time-point (“12 days after sorting” in Figure 4I and Figure S6G) were re-plated on 12-well plates (100,000 cells per well) and used for RNA-extraction 12 days later.

- Alkaline Phosphatase (AP) staining for reprogramming efficiency calculation

Reprogramming cell cultures were subjected to AP staining for the identification of iPSC colonies as described before [10]. In brief, cells growing on plates were washed with PBS and fixed with 4% formaldehyde at 4 °C for 10 min. The cells were washed with NTMT buffer [100 mM Tris-HCl, 100 mM NaCl, 50 mM MgCl_2_ and 0.1% Tween-20, pH 9.5] and then stained for AP activity, using NBT/BCIP substrate solution (Roche Life Science, Marousi, Greece, 11681451001) diluted 1/100 in NTMT buffer. Reprogramming efficiency was calculated by dividing the number of AP(+) iPSC colonies with the total number of cells that were seeded on the feeder-covered plates and then by multiplying by 100. Reprogramming efficiency evaluation was performed in biological triplicates.

- RNA isolation and real-time PCR

Cells were lysed with NucleoZOL (Macherey-Nagel, Dueren, Germany, 740404) and their RNA was extracted following the manufacturer’s instructions. Reverse transcription followed to produce cDNA with the PrimeScript™ RT Reagent Kit (Perfect Real Time) (TaKaRa Bio, Shiga, Japan, RR037A) using oligodT primers and random 6mers. The cDNA quantification was performed in the CFX96 Touch™ Real-Time PCR Detection System (Bio-Rad, Hercules, CA, USA) using the 2x KAPA SYBR^®^ FAST Universal mix (KAPA Biosystems, Roche Life Sciences, Marousi, Greece, KK4618) and the appropriate primer pair (Table S7). Calculation of expression values was performed with the 2^(−ΔCt) method and *Gapdh* served as the reference gene. *Gfp* expression values of the enhancer reporters in Figure 4C and Figure S6C,D were normalized to the *Gfp* values of LeGO-G2 at the respective time-points, to compensate for putative fluctuations in *Gfp* expression caused by the intense epigenetic alterations occurring in reprogramming at the random chromatin integration sites of the reporters.

#### 4.1.8. RNA Sequencing

- Reprogramming time-course

“4F-MEFs” (day 0) and cells undergoing reprogramming were isolated at different time-points (day 1, day 2, day 3, day 4, day 6, day 8) and their RNA was extracted using NucleoZOL (Macherey-Nagel, Dueren, Germany, 740404), as by the manufacturer’s instructions. Two biological replicates were prepared for each sample. The RNAs were DNAseI-treated and cleaned up using the classic phenol–chloroform procedure. RNA-seq experiments were carried out in the Greek Genome Center (GGC) of the Biomedical Research Foundation of the Academy of Athens (BRFAA). RNA-seq libraries were prepared using the NEBNext^®^ Ultra II™ Directional RNA Library prep Kit for Illumina^®^ (NEB, Ipswich, MA, USA, E7760) with 1 ug of total RNA input. Library QC was performed with the Agilent Bioanalyzer DNA 1000 kit (Agilent, Santa Clara, CA, USA) and quantitation with the Qubit™ High Sensitivity (HS) spectrophotometric method (Invitrogen, Thermo Fisher Scientific, Waltham, MA, USA). Sequencing was performed in the Illumina NovaSeq 6000 sequencer (Illumina, San Diego, CA, USA) and we obtained on average 22 × 10^6^ single-end, reverse-stranded, 101 bp-long reads per sample. The data of the RNA-seq experiment are deposited in the Gene Expression Omnibus data repository under the GEO accession ID GSE274132.

- Upp1_800_-GFP sorted cells

GFP(+) and GFP(-) cells collected after sorting (as described above) were subjected to RNA extraction using the NucleoSpin RNA kit (Macherey-Nagel, Dueren, Germany, 740955). The DNA was removed by a DNAseI on-column step, as instructed by the kit’s manual. The library preparation and sequencing was performed as described above. Two biological replicates were sequenced per sample. The data of the RNA-seq experiment are deposited in the Gene Expression Omnibus data repository under the GEO accession ID GSE279976.

### 4.2. Bioinformatics Analyses

#### 4.2.1. OSKM ChIP-Seq Data Analysis

- Selection and primary analysis of published O/S/K/M and Nanog ChIP-seq datasets

For our O/S/K/M ChIP-seq analysis, we utilized multiple datasets derived either from our laboratory (current publication and [10]), or by others [16,17,18,37], deposited under the GEO IDs GSE114581, GSE274131 GSE90893, GSE67520, GSE101905 and GSE113429 (Table S1). We selected datasets that use the 4-factor reprogramming system (OKSM) and MEFs as the starting population. Prior to merging with other datasets, we re-analyzed our O/S/K/M ChIP-seq data under the GEO ID GSE114581 [10], wishing to take advantage of the latest adjustments to the existing algorithms. In more detail, Fastq files were aligned against the mm10 mouse genome with Bowtie2 aligner [83] and the output files were filtered for low-quality reads, duplicates, blacklist regions and mitochondrial genome entries with custom-made pipeline from BEDtools [84] and SAMtools [85]. Peak calling was performed with the MACS2 callpeak command [86]. Bigwig files were constructed with the deepTools bamCompare tool [87], using the “subtract“ option to remove Input signal from the respective immunoprecipitation (IP) signal, and choosing RPKM for normalization. The same pipeline was performed for our Nanog ChIP-seq experiment analysis, but first, the quality of the Fastq files was examined with the FastQC software (v0.12.1) [88]. Finally, for the production of bigwig files for Figure 3 and Figure S4, raw data of the O/S/K/M published datasets that were used in this study (GSE90893, GSE67520, GSE101905, GSE113429) were downloaded from the SRA database and analyzed as described above.

- Merging of datasets

Our O/S/K/M ChIP-seq dataset (GSE114581) was re-analyzed as described above, while the other ChIP-seq peaks were used as published (GSE90893, GSE67520, GSE101905, GSE113429) (see Table S1 for more details). The peaks (bed files) were combined per time-point for each individual transcription factor (using cat and sort functions) and overlaps between peaks from different datasets were combined using the BEDtools mergeBed tool (option “-d 300”) [84]. For the day 1 time-point, we combined the 18 h and day 1 data, and for the day 6 time-point, we combined the day 5 and day 6 data. We also combined data from both high- (SSEA1+) and low- (Thy1+) efficiency cells when available, since we are interested in studying the bulk population of cells undergoing reprogramming. For the analysis of OSKM binding as a group (Figure 1E and Figure 2), we merged the Oct4, Sox2, Klf4 and Myc peaks per time-point, as we did above (cat, sort, mergeBed -d 300). The peaks of the combined dataset are presented in Table S2.

For the visualization of the combined dataset in the Integrative Genomics Viewer (IGV) [89] (Figure 3 and Figure S4), we used the normalized bigwig files from each dataset, as produced by the analysis described above. Then, the different bigwig files were merged and averaged for each factor per time-point, using the WiggleTools mean tool [90].

#### 4.2.2. Histone Modifications ChIP-Seq Analysis

The histone modifications ChIP-seq bioinformatics analyses were performed using already published data (GEO ID GSE90893 [17]) and The Galaxy Suite platform [91]. The quality of the sequencing reads was evaluated using the FastQC algorithm (v0.12.1) [88]. Reads were trimmed to 50 bp using the Trimmomatic tool [92]. Sequencing reads were mapped to the mm9 version of the mouse genome using the Bowtie2 algorithm with the “very sensitive end to end” option [83]. Duplicates were removed with the RmDup command from Samtools [85]. Peaks were called using the MACS2 algorithm with a q-value cut-off of 0.05 [86]. Bigwig files were constructed using the bamCoverage command from the deepTools suite [93] with RPKM as the normalization method.

#### 4.2.3. ATAC-Seq Analysis

ATAC-seq bioinformatics analyses were performed using The Galaxy Suite [91]. The quality of the sequencing reads was evaluated using the FastQC algorithm (v0.12.1) [88]. Reads were trimmed to 50 bp using the Trimmomatic tool [92]. Sequencing reads were mapped to the mm10 version of the mouse genome using the Bowtie2 algorithm with the “very sensitive end to end” option [83]. The Samtools Fixmate command was used [85] prior to duplicate removal with the MarkDuplicates command from Picard tools using the option “do not write duplicates to the output file”. Reads from all samples were downsampled to the same sequencing depth using the Downsample BAM option from Picard tools. Peaks were called using the MACS2 algorithm with the options “no model”, “extension-200”, “shift-100” and a q-value cut-off of 0.05 [86]. Bigwig files were constructed using the bamCoverage command from the deepTools suite [93] with RPKM as the normalization method.

#### 4.2.4. Investigation of OSKM Binding Sites

- Calculation of common binding sites between Oct4, Sox2, Klf4 and Myc

For the analysis presented in Figure 1B–D, we utilized our combined dataset after downsampling the Oct4 day 1, day 3 and day 6 datasets. This was necessary in order to avoid a bias over Oct4 overlaps, due to the higher number of Oct4 peaks in our combined dataset. We downsampled randomly the number of Oct4 peaks to match the average number of the Sox2 and Klf4 peaks of the respective time-point, using a custom-made pipeline (sed command). For the calculation of the common sites between each pair of the OSKM factors we used BEDTools intersect (-wa -u options) [84].

- Identification of ESC, transient and MEF sites

For this analysis (Figure 1E and Figure 2), we used the merged OSKM peaks and not the individual Oct4, Sox2, Klf4 and Myc sites (see ChIP-seq analysis above). All sites where OSKM were present at the ESC stage were considered as “ESC OSKM binding sites”. For the identification of the “early-bound” and “late-bound” categories of ESC sites presented in Figure 1E and Figure 2A, we performed intersection of all the ESC sites with the day 1 OSKM binding sites using the BEDTools intersect tool (-wa -u and -v options, respectively) [84]. For the early-bound ESC subcategories in Figure 2I–L, we performed successive intersections as described above (-wa -u or -v options), with the day 3 and day 6 OSKM binding sites, accordingly. The same was performed for the identification of MEF “pre-bound” and “de novo sites” presented in Figure 3A.

For the OSKM “Transient sites” (Figure 1E), we followed a similar logic of successive intersections, as described above, in order to combine all the different categories of transiently-bound sites (sites where OSKM bind at least at one intermediate time-point, but neither in MEFs, nor in ESCs). In more detail, for the transient sites where OSKM bind only at one time-point (i.e., day 1, day 3, or day 6), we removed (-v option) from the respective bed files (i.e., the total day 1, day 3, or day 6 OSKM peaks) the overlaps with the ESC OSKM sites and, subsequently, the overlaps with the other two remaining intermediate time-points (i.e., we removed the day 3-day 6, day 1-day 6 and day 1-day 3 OSKM peaks, respectively). Finally, we removed the common peaks of the resulting files with the KM MEF binding sites. The remaining sites were considered the day 1-, day 3- and day 6-only transient OSKM sites. The transient sites where OSKM bind at two intermediate time-points (i.e., day 1-day 3, day 1-day 6, and day 3-day 6 sites) were calculated by pairwise intersections of the respective time-point OSKM peaks. In each case, for every overlap, we kept the original sites of both intersected files and merged them (cat, sort and mergeBed with default options). Then, we removed the overlaps with the remaining intermediate time-point (i.e., day 6, day 3 or day 1, respectively), with ESCs and subsequently with MEFs. Finally, for the transient sites where OSKM bind at all intermediate time-points (day 1-day 3-day 6 sites), we intersected in a stepwise manner the three respective OSKM files and for every overlap we kept and merged the original sites of all intersected files. From the resulting file, we kept only the sites where OSKM were not also present in ESCs and MEFs. The final file containing all “Transient sites” (shown in Figure 1E) was produced by merging all the above categories of sites (cat, sort and mergeBed with default options).

For the “MEF sites, lost at ESCs” (Figure 1E), we combined the different categories of sites where (OS)KM bind at MEFs but not in ESCs. In more detail, the sites bound only at MEFs were calculated by keeping the KM MEF peaks that did not overlap with ESCs and neither with any of the other time-points in reprogramming. The rest of the MEF sites, which were lost progressively in reprogramming, were calculated by merging all the Transient site files before the removal of the overlaps to the MEF peaks and intersecting them with the MEF KM peaks. The final file containing all “MEF sites, lost at ESCs” (shown in Figure 1E) was produced by merging the two above categories of sites (cat, sort and mergeBed with default options).

Using multiIntersect tool, we ensured that the identified (sub)categories of the ESC, Transient and MEF OSKM-bound sites did not overlap with each other. Thus, all OSKM sites presented in this study belong to a single (sub)category of sites.

- Overlaps of OSKM sites with ESC Super-enhancers

For the calculation of overlaps between the ESC super-enhancers [40] and the various types of OSKM ESC sites presented in Table S4, we used the BEDTools intersect tool, as above (-wa -u and -c options) [84].

- Epigenetic characterization of the OSKM binding sites

The percentage of statistically significant ATAC-open and ATAC-closed chromatin regions (heatmaps and Venn diagrams in Figure 2A,I–L) was calculated using the BEDTools intersect tool for each category of sites and for all ATAC-seq examined time-points. The ATAC-seq and histone modifications summary plots and heatmaps were constructed with the plotHeatmap tool of deepTools (v3.5.2) [87]. The matrices that were used as input for the plotHeatmap were calculated with computeMatrix reference-point mode and the options “--referencePoint center” “--averageTypeBins mean”, “--missingDataAsZero”, and “-bs 5”. For the depiction of the histone modifications ChIP-seq experiments (Figure S1), the examined OSKM sites were converted to the mm9 mouse genome assembly.

- Gene assignment to OSKM binding sites

Assignment of the various OSKM binding sites to genes was performed with GREAT online tool (v4.0.4) [94,95] using the whole genome as background and the “Single nearest gene” option for 1000 kb maximum distance. For the calculation of common gene sets between the different OSKM ESC site subcategories (Figure S2, Table S3), we used the Venny tool (v2.1) [96].

- Motif analysis

Our de novo motif analysis was performed with HOMER [97]. We used the options “-size given” and “-preparse”. The motifs presented in this paper have a final enrichment *p*-value < 1 × 10^−10^, appear at least at 5% of the target sequences and have a score ≥ 0.9.

#### 4.2.5. Method for the Identification of Putative Reprogramming-Inducible Enhancers

To identify Reprogramming-Inducible Enhancers (RIEs) in the mouse genome, we used the de-novo-acquired early-bound ESC sites (Figure 3A, right panel). Using GREAT (v4.0.4) [94,95] and the “Single nearest gene” option for 2.5 kb maximum distance, we found that 2865 genes are associated with the above regions. Among them, we chose only the sites close to the 610 genes that are up-regulated between MEFs and ESCs, with Log_2_FC > 2 and p-adjusted < 0.05 (702 sites). Then, we intersected the resulting sites with all the individual Oct4, Sox2, Klf4 and Myc peaks in reprogramming using BEDTools (-c option) [84] to identify and remove regions with a low number of binding events (≤10 peaks). The remaining 66 sites were considered as putative RIE elements (Table S5).

#### 4.2.6. RNA-Seq Analysis

The quality of the sequenced samples was evaluated by the FastQC tool (v0.12.1) [88]. Then, the FASTQ files were aligned to the UCSC mm10 genome version with the HISAT2 tool (v2.1.0) using the options “-5 4 -3 4” to trim the 5′ and 3′ ends of the reads and “--rna-strandness R” [98]. Annotation of the SAM files to produce the raw counts was performed with the HTSeq htseq-count tool (v0.6.1p1 and 2.0.3) [99] and the options “-s reverse” “-m intersection-nonempty”, using the UCSC mm10refGene.gtf genome annotation file. Normalized counts were calculated using DESeq2 (v1.30.1, estimateSizeFactors) [100]. To calculate the exogenous *Gfp* gene’s expression counts and Log_2_FC between the Upp1_800_-GFP(+) and GFP(−) cells, we performed alignment of the Fastq files to a custom mm10 genome file containing the Upp1_800_-GFP transgene sequence and the annotation of the SAM files with a custom .gtf file containing the *Gfp* gene entry. To calculate the normalized counts for the reprogramming time-course, we used also the raw counts of the sample “ESCs wild type” as they are uploaded in the GEO database (GSE215883, Table S1). Genes with zero normalized counts at all time-points were excluded when calculating the median expression value of multiple genes. DEGs were considered the genes with Log_2_FC > 0.58 or <−0.58 and p-adjusted < 0.05.

#### 4.2.7. Single-Cell RNA-Seq Analysis

Single-cell RNA-seq analysis was performed at samples derived from the Schiebinger et al., 2019 dataset [60]. Matrix, genes and barcode files for day 0, day 2, day 4, day 6, day 8 and iPSCs were downloaded from GEO database (GSE122662). Analysis was performed with the “Seurat” package (v4) [101]. Briefly, cells with mitochondrial counts > 5% were filtered, and the remaining cells were scaled using the “LogNormalize” method and “ScaleData” command, respectively, while variable features were identified using the “FindVariableFeatures” command. For the analyzed datasets, we found the Integration Anchors with the function “FindIntegrationAnchors” and integrated all the data in a common Seurat Object with the function “IntegrateData”. Subsequently, we performed data normalization (“NormalizedData” function), we found variable features (“FindVariableFeatures” function), we performed data scaling (“ScaleData” function) and finally, we performed UMAP visualization (“RunUMAP” function”) using as input the PCA (“RunPCA” function). We plotted *Upp1* expression among the cell populations using “FeaurePlot” function, while co-expression analyses were performed using “FeaturePlot” function with the option “blend=TRUE”.

#### 4.2.8. Functional Enrichment of Gene Sets

For the functional enrichment analyses presented in Figure 2D,G and Figure 4G we used Webgestalt [102], while the analyses in Figure S2 were performed with EnrichR [103,104,105]. Finally, for the networks analysis in Figure S7 we utilized the STRING Enrichment tool of the stringApp [106] in Cytoscape [107]. We utilized the libraries GO Biological Process 2023 and GO Molecular Function 2023 [108,109], KEGG Pathways [110,111,112], Reactome Pathways [113], MSigDB Hallmark 2020 [114,115] and PanglaoDB Augmented 2021 [116].

#### 4.2.9. Network Analysis

For the network analysis of the day 2 Upp1_800_-GFP(+)/(−) RNA-seq dataset presented in Figure S7, we inserted all the DEGs (Log_2_FC > 0.58 or <−0.58 and p-adjusted < 0.05) in Cytoscape STRING protein query [107] to create a “full STRING network” with confidence cut-off 0.4. Then, the network was clustered with the MCODE application [51] using the default parameters and without the “haircut” option. The Log_2_FC value of each DEG node was assigned to a blue–red color gradient (blue for down-regulated genes, red for up-regulated genes). The color scale has the same minimum and maximum values for all networks appearing in Figure S7.

#### 4.2.10. Other Graphical Representations

The Ggplot2 package [117] was used in R (v4.0.5) for the construction of the gene expression line-plots, the functional enrichment and motif analyses dot-plots, the PCA and the volcano plots. All heatmaps were designed using the pheatmap package in R. Statistical analyses and plots in Figure 4 and Figure S6 were performed using GraphPad Prism version 8.0.2 for Windows, GraphPad Software, Boston.

## Data Availability

The new data produced and presented in the study (raw and analyzed files) are included in the Supplementary Materials and/or are openly available in the Gene Expression Omnibus data repository under the GEO accession IDs GSE274130, GSE274131, GSE274132 and GSE279976. Already published data used in the study (either analyzed again, or used as published) are available in the Gene Expression Omnibus data repository under the GEO accession IDs GSE114581, GSE90893, GSE67520, GSE101905, GSE113429, GSE215883 and GSE122662. A detailed list of all the datasets used in this study is also available in the Supplementary Materials (Table S1).

## Supplementary Materials

The following supporting information can be downloaded at: https://www.mdpi.com/article/10.3390/ijms252313128/s1.
