# Supplementary material for: An Unbiased Approach to Identifying Cellular Reprogramming-Inducible Enhancers
Source: Int J Mol Sci. 2024 Dec 6;25(23):13128. doi: 10.3390/ijms252313128 (PMC11642860; doi:10.3390/ijms252313128)
Supplement: Supplementary file 1 [file ijms-25-13128-s001.zip › Supplementary-tables_legends.pdf]

International Journal of
Molecular Sciences

MDPI

Supplementary Tables Legends

Table S1. The datasets used in the present study.

Table S2. Combined dataset ChIP-seq peaks for OSKM and Nanog (mm10).

Table S3. Genes associated with the identified OSKM-bound sites using the GREAT algorithm (Referred to Figures 2I and S2)**.**

Table S4. Overlap of the ESC Super-enhancers with the identified OSKM-bound sites. Shown are the total number of overlaps of the OSKM-bound ESC sites with the ESC Super-enhancers (Whyte et al, 2013). The green-color heatmap indicates the number of overlaps with the ESC Super-enhancers (dark green corresponds to higher number of overlaps).

Table S5. List of the identified putative Reprogramming-Inducible Enhancers (RIEs). Shown are the 66 putative RIEs (mm10), along with the number of O/S/K/M peaks detected on them, the genes associated with each of the RIEs, their distance from the gene's TSS and the Log_2FC and p-adjusted values of the respective gene's expression difference between ESCs and MEFs. Also, for each putative RIE element we show the number of overlaps with the ESC-specific super-enhancers and the ESC MTL sites.

Table S6. Day 2 $Upp1_{800}$-GFP(+) and GFP(-) RNA-seq dataset (normalized counts and DEGs list) (Referred to Figure 4, S6F and S7)**.**

Table S7. Primers used in the present study and DNA sequences of the cloned RIEs.
