# Supplementary material for: An Unbiased Approach to Identifying Cellular Reprogramming-Inducible Enhancers
Source: Int J Mol Sci. 2024 Dec 6;25(23):13128. doi: 10.3390/ijms252313128 (PMC11642860; doi:10.3390/ijms252313128)
Supplement: Supplementary file 1 [file ijms-25-13128-s001.zip › Supplementary_figures.pdf]

Figure S1

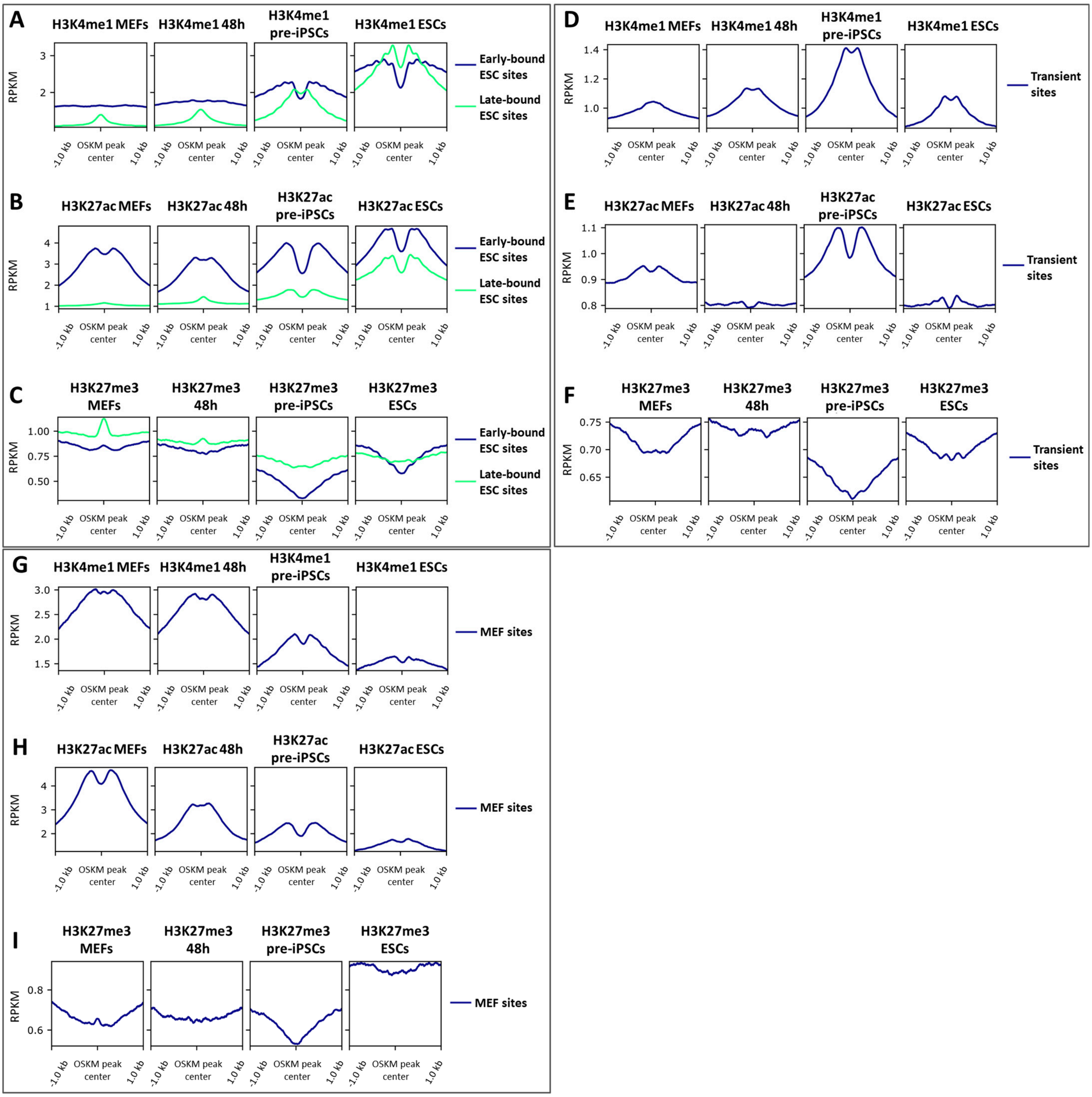

Figure S1. Epigenetic marks at the different classes of OSKM binding sites during cellular reprogramming. (Referred to Figure 1E). (**A**) Summary plots depicting the H3K4me1 ChIP-seq signal (data taken from Chronis et al., 2017) at the early- and late-bound OSKM ESC sites from -1kb to +1km from the center of the OSKM peaks during cellular reprogramming (MEFs, 48h, pre-iPSCs, ESCs). The signal is calculated as RPKM.; (**B**) As in A, but for the H3K27ac histone modification at the early- and late-bound OSKM ESC sites.; (**C**) As in A, but for the H3K27me3 histone modification at the early- and late-bound OSKM ESC sites.; (**D**) As in A, but for the H3K4me1 histone modification at the transient OSKM sites.; (**E**) As in A, but for the H3K27ac histone modification at the tran-

sient OSKM sites.; (**F**) As in A, but for the H3K27me3 histone modification at the transient OSKM sites.; (**G**) As in A, but for the H3K4me1 histone modification at the (OS)KM MEF sites.; (**H**) As in A, but for the H3K27ac histone modification at the (OS)KM MEF sites.; (**I**) As in A, but for the H3K27me3 histone modification at the (OS)KM MEF sites.

Figure S2

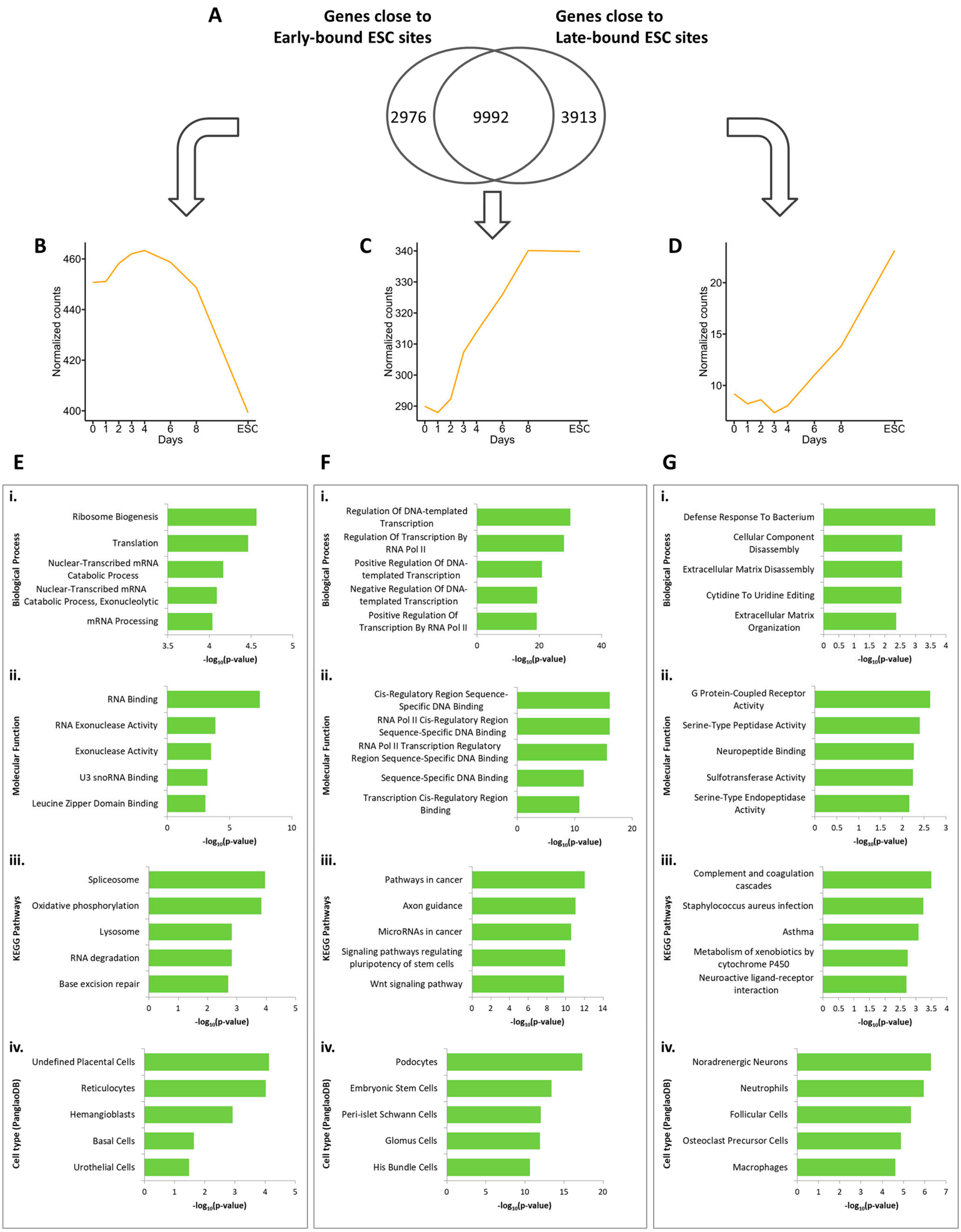
A
Genes close to Early-bound ESC sites
Genes close to Late-bound ESC sites
2976
9992
3913
B
C
D
Normalized counts
Days
ESC
E
F
G
i.
ii.
iii.
iv.
Biological Process
Molecular Function
KEGG Pathways
Cell type (PanglaoDB)
-log10(p-value)
Ribosome Biogenesis
Translation
Nuclear-Transcribed mRNA Catabolic Process
Nuclear-Transcribed mRNA Catabolic Process, Exonucleolytic
mRNA Processing
RNA Binding
RNA Exonuclease Activity
Exonuclease Activity
U3 snoRNA Binding
Leucine Zipper Domain Binding
Spliceosome
Oxidative phosphorylation
Lysosome
RNA degradation
Base excision repair
Undefined Placental Cells
Reticulocytes
Hemangioblasts
Basal Cells
Urothelial Cells
Regulation Of DNA-templated Transcription
Regulation Of Transcription By RNA Pol II
Positive Regulation Of DNA-templated Transcription
Negative Regulation Of DNA-templated Transcription
Positive Regulation Of Transcription By RNA Pol II
Cis-Regulatory Region Sequence-Specific DNA Binding
RNA Pol II Cis-Regulatory Region Sequence-Specific DNA Binding
RNA Pol II Transcription Regulatory Region Sequence-Specific DNA Binding
Sequence-Specific DNA Binding
Transcription Cis-Regulatory Region Binding
Pathways in cancer
Axon guidance
MicroRNAs in cancer
Signaling pathways regulating pluripotency of stem cells
Wnt signaling pathway
Podocytes
Embryonic Stem Cells
Peri-islet Schwann Cells
Glomus Cells
His Bundle Cells
Defense Response To Bacterium
Cellular Component Disassembly
Extracellular Matrix Disassembly
Cytidine To Uridine Editing
Extracellular Matrix Organization
G Protein-Coupled Receptor Activity
Serine-Type Peptidase Activity
Neuropeptide Binding
Sulfotransferase Activity
Serine-Type Endopeptidase Activity
Complement and coagulation cascades
Staphylococcus aureus infection
Asthma
Metabolism of xenobiotics by cytochrome P450
Neuroactive ligand-receptor interaction
Noradrenergic Neurons
Neutrophils
Follicular Cells
Osteoclast Precursor Cells
Macrophages

Figure S2. OSKM early- and late-bound ESC genes (Referred to Figure 2A-G). (**A**) Venn diagram depicting the number of common OSKM target genes between the early- and late-bound ESC sites.; (**B**) Shown is a line graph depicting normalized counts of expression of the genes associated with the OSKM early-bound ESC sites, only. The median expression value is depicted for each time-point.; (**C**) As in B, but for the genes associated with both the OSKM early- and the late-bound ESC sites.; (**D**) As in B, but for the genes associated with the OSKM late-bound ESC sites, only.; (**E**) Shown are bar graphs indicating the Functional enrichment analysis (Over-representation analysis, ORA) for the genes associated with the OSKM early-bound ESC sites, only, as defined from the analysis used in A. Depicted are the top 5 terms sorted by p-value (p-value<0.01). The libraries GO Biological Process 2023, GO Molecular Function 2023, KEGG Pathways 2021, and PanglaoDB Augmented 2021 are used.; (**F**) As in E but for the genes associated with both the OSKM early- and late-bound ESC sites (p-value<0.01).; (**G**) As in E, but for the genes associated with the OSKM late-bound ESC sites, only (p-value<0.01).

Figure S3

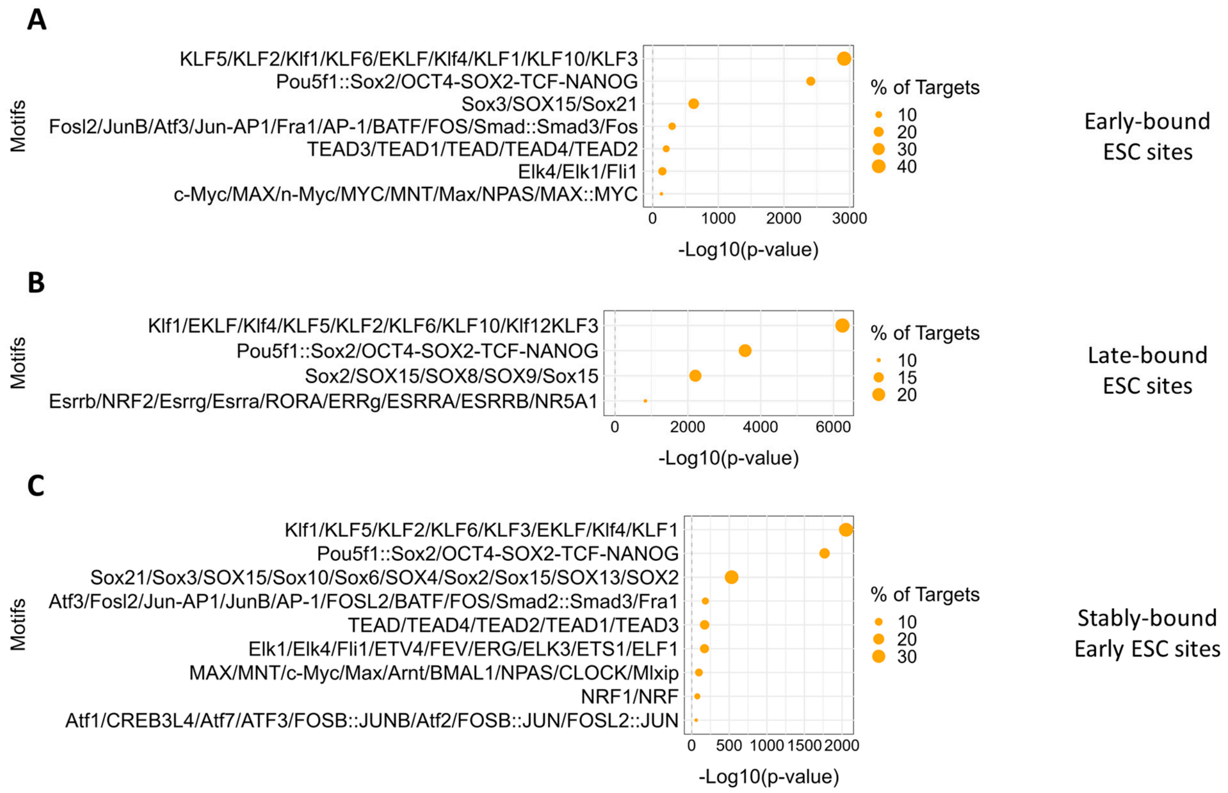

Figure S3. Motif analysis of the OSKM ESC genomic sites (Referred to Figure 2A and 2I). (**A**) De novo motif analysis of the early-bound ESC sites. Depicted are the motifs obtained with a final enrichment p-value<1e-10. Only motifs with score≥0.9 were considered. The grey dashed line corresponds to p-value=0.01.; (**B**) As in A, but for the late-bound ESC sites.; (**C**) As in A, but for the stably-bound early ESC sites.

Figure S4

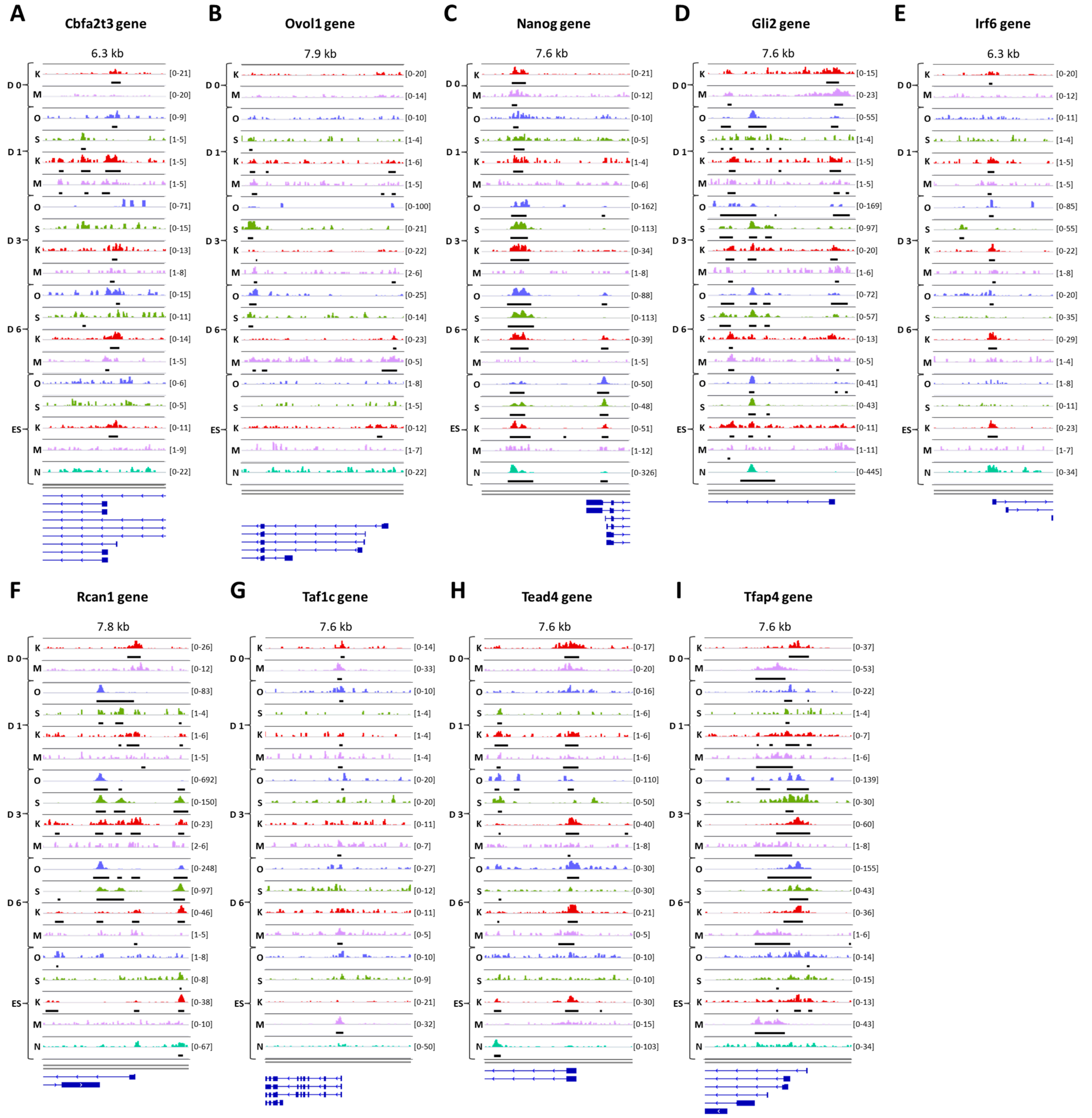

Figure S4. OSKM and Nanog binding to the 9TR-GRN loci (adapted from Papathanasiou et al., 2021). (**A-I**) Shown are ChIP-seq bigwig files in the IGV browser depicting the binding of Oct4 (blue), Sox2 (green), Klf4 (red), Myc (purple) and Nanog (cyan) to putative regulatory regions in the 9TR-GRN genes in MEFs undergoing reprogramming (day 1, day 3 and day 6), in control MEFs (day 0) and ESCs. The scale for each snapshot is shown on the right. The binding signal has been calculated as RPKM, after subtraction of the Input signal from the respective IP signal. Statistically significant peaks are depicted with a black bar below the respective lane.

Figure S5

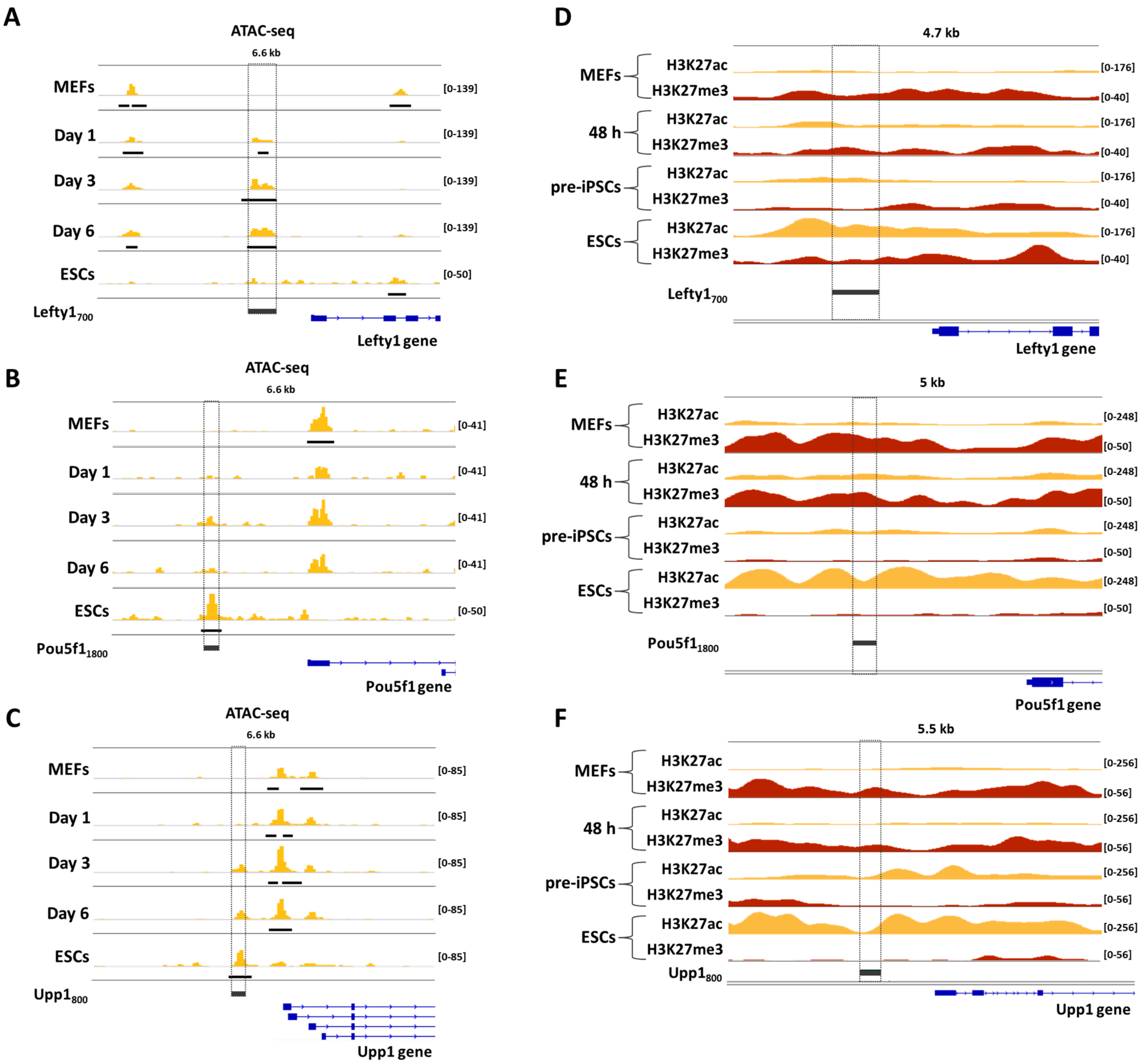

Figure S5. Chromatin structure at the Lefty1$_{700}$, Pou5f1$_{1800}$ and Upp1$_{800}$ elements during cellular reprogramming. (**A**) Shown are ATAC-seq bigwig files in the IGV browser depicting chromatin accessibility at the Lefty1$_{700}$ RIE in MEFs undergoing reprogramming (day 1, day 3 and day 6), in control MEFs and ESCs. The scale for each snapshot is shown on the right. Statistically significant peaks are depicted with a black bar below the respective lane. The position of the Lefty1$_{700}$ element is depicted as a dark grey bar at the bottom of the panel. The signal is calculated as RPKM; (**B**) As in A, but for the Pou5f1$_{1800}$ element.; (**C**) As in A, but for the Upp1$_{800}$ element.; (**D**) Shown are ChIP-seq wig files in the IGV browser depicting the H3K27Ac (yellow) and H3K27me3 (dark red) at the Lefty1$_{700}$ putative RIE in MEFs and during reprogramming (data taken from Chronis et al., 2017). The scale for each snapshot is shown on the right. The position of the Lefty1$_{700}$ element is depicted as a dark grey bar at the bottom of the panel.; (**E**) As in D, but for the Pou5f1$_{1800}$ element.; (**F**) As in D, but for the Upp1$_{800}$ element.

Figure S6

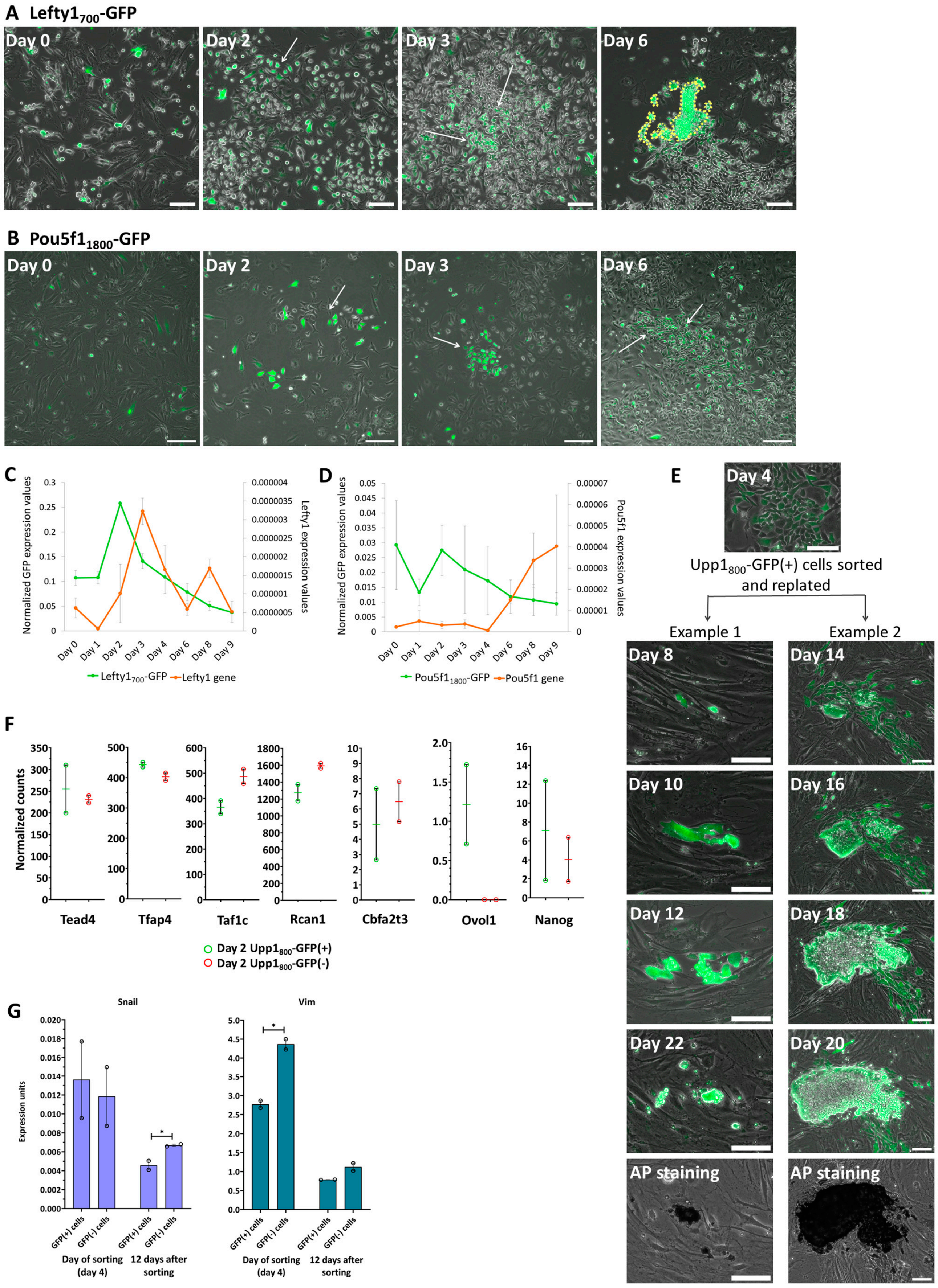
A Lefty1700-GFP
Day 0
Day 2
Day 3
Day 6
B Pou5f11800-GFP
Day 0
Day 2
Day 3
Day 6
C
Normalized GFP expression values
Lefty1 expression values
Day 0
Day 1
Day 2
Day 3
Day 4
Day 6
Day 8
Day 9
Lefty1700-GFP
Lefty1 gene
D
Normalized GFP expression values
Pou5f1 expression values
Pou5f11800-GFP
Pou5f1 gene
E
Day 4
Upp1800-GFP(+) cells sorted and replated
Example 1
Example 2
Day 8
Day 14
Day 10
Day 16
Day 12
Day 18
Day 22
Day 20
AP staining
AP staining
F
Normalized counts
Tead4
Tfap4
Taf1c
Rcan1
Cbfa2t3
Ovol1
Nanog
Day 2 Upp1800-GFP(+)
Day 2 Upp1800-GFP(-)
G
Snail
Vim
Expression units
GFP(+) cells
GFP(-) cells
Day of sorting (day 4)
12 days after sorting

Figure S6. The Lefty1$_{700}$ and Pou5f1$_{1800}$ identified putative Reprogramming-Inducible Enhancers (RIEs) and additional data for the Upp1$_{800}$-GFP(+) and GFP(-) cells during cellular reprogramming. (**A**) Representative fluorescence microscopy images taken from a time-course reprogramming experiment using MEFs transduced with the lenti-virus bearing the Lefty1$_{700}$-GFP reporter cassette. The brightfield (phase contrast) and fluorescence images were merged for each time-point. The white arrows point to cells abandoning the MEF phenotype (MET). Early iPSC formations are indicated with yellow dashed lines. Scale bar: 150μm.; (**B**) As in A, but for the Pou5f1$_{1800}$-GFP reporter. Scale bar: 200μm.; (**C**) Line graph depicting the expression of the endogenous *Lefty1* gene (orange line, right axis) in comparison with the expression of the Lefty1$_{700}$-GFP transgene (green line, left axis). Shown are the mean expression values from two biological replicates and the standard error.; (**D**) As in C, but for the Pou5f1$_{1800}$ element and the *Pou5f1* gene.; (**E**) Representative fluorescence microscopy images taken from a time-course reprogramming experiment using MEFs transduced with the lenti-virus bearing the Upp1$_{800}$-GFP reporter cassette, as indicated. The GFP(+) cells were isolated on day 4 of reprogramming and after re-plating were left to continue the reprogramming process. The images depict the same region of the plate as it develops to iPSCs during the reprogramming process. The brightfield (phase contrast) and fluorescence images have been merged for each picture. Scale bar: 100μm.; (**F**) Dot plots depicting normalized counts for the expression of *Tead4*, *Tfap4*, *Taf1c*, *Rcan1*, *Cbfa2t3*, *Ovol1* and *Nanog* genes in the Upp1$_{800}$-GFP(+) and GFP(-) cells, as calculated by RNA-seq on day 2 of reprogramming. Two biological replicates are depicted. The mean and the standard error are also depicted.; (**G**) Bar-chart depicting the relative expression units of *Snail* and Vimentin (*Vim*) in the Upp1$_{800}$-GFP(+) and GFP(-) on day 4 (day of isolation) and 12 days after isolation of the cells. Two biological replicates are depicted for each cell type. The mean and the standard error are also depicted. Unpaired two-tailed t-test was performed and the statistically significant differences are represented on the figure (*Snail*-12 days after sorting: $t=4.328$, $df=2$, p-value=0.0495, *Vim*-day 4: $t=9.560$, $df=2$, p-value=0.0108). *: p-value < 0.05

Figure S7

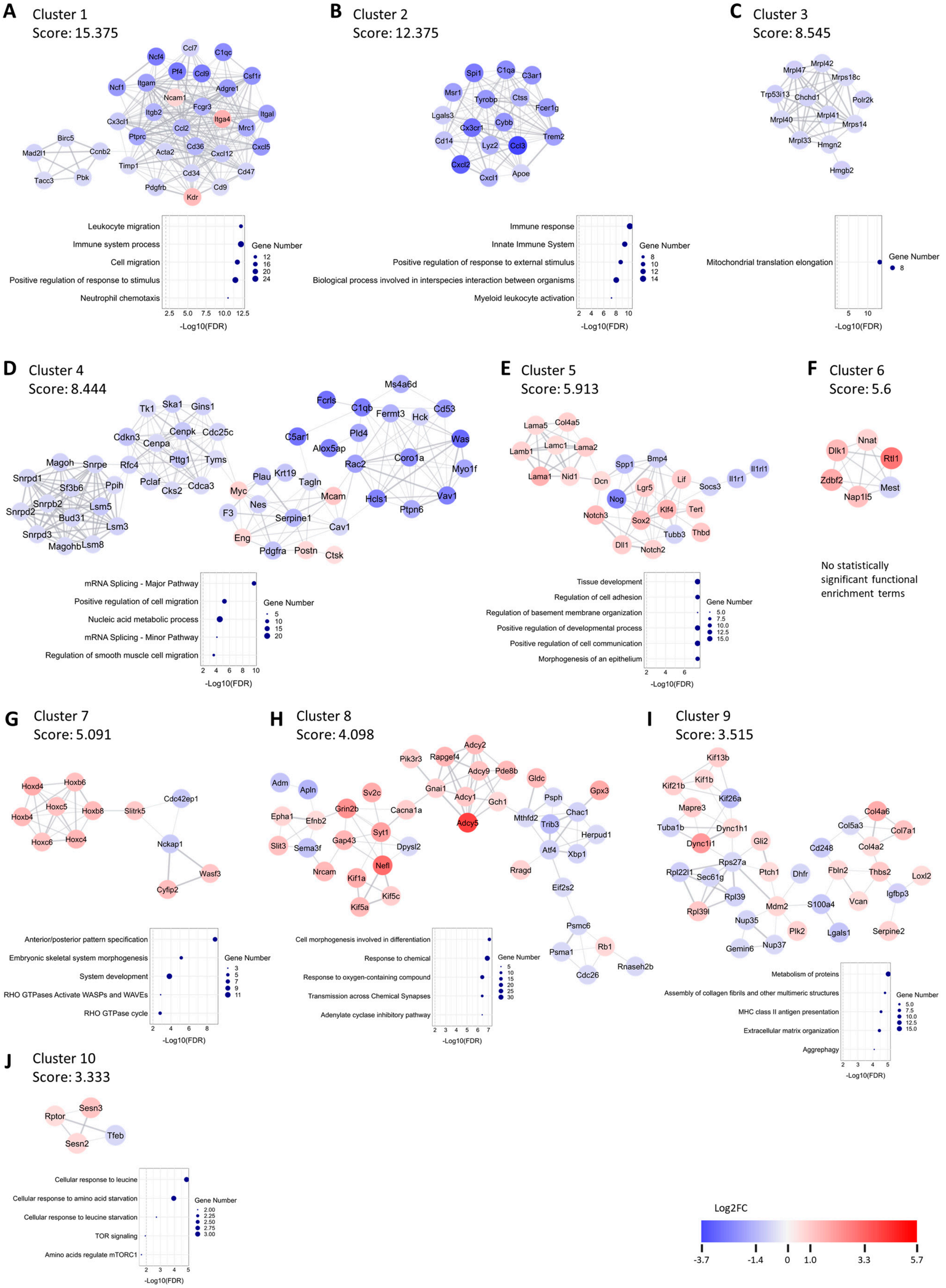
A Cluster 1
Score: 15.375
Leukocyte migration
Immune system process
Cell migration
Positive regulation of response to stimulus
Neutrophil chemotaxis
Gene Number
-Log10(FDR)
B Cluster 2
Score: 12.375
Immune response
Innate Immune System
Positive regulation of response to external stimulus
Biological process involved in interspecies interaction between organisms
Myeloid leukocyte activation
C Cluster 3
Score: 8.545
Mitochondrial translation elongation
D Cluster 4
Score: 8.444
mRNA Splicing - Major Pathway
Positive regulation of cell migration
Nucleic acid metabolic process
mRNA Splicing - Minor Pathway
Regulation of smooth muscle cell migration
E Cluster 5
Score: 5.913
Tissue development
Regulation of cell adhesion
Regulation of basement membrane organization
Positive regulation of developmental process
Positive regulation of cell communication
Morphogenesis of an epithelium
F Cluster 6
Score: 5.6
No statistically significant functional enrichment terms
G Cluster 7
Score: 5.091
Anterior/posterior pattern specification
Embryonic skeletal system morphogenesis
System development
RHO GTPases Activate WASPs and WAVEs
RHO GTPase cycle
H Cluster 8
Score: 4.098
Cell morphogenesis involved in differentiation
Response to chemical
Response to oxygen-containing compound
Transmission across Chemical Synapses
Adenylate cyclase inhibitory pathway
I Cluster 9
Score: 3.515
Metabolism of proteins
Assembly of collagen fibrils and other multimeric structures
MHC class II antigen presentation
Extracellular matrix organization
Aggrephagy
J Cluster 10
Score: 3.333
Cellular response to leucine
Cellular response to amino acid starvation
Cellular response to leucine starvation
TOR signaling
Amino acids regulate mTORC1
Log2FC
-3.7
-1.4
0
1.0
3.3
5.7

Figure S7. Dense subnetworks of the DEGs from the day 2 $Upp1_{800}$-GFP(+) and GFP(-) cells' RNA-seq. (**A-J**) Reconstruction of clusters extracted from the whole DEG network assembled from the day 2 $Upp1_{800}$-GFP(+) cells, as compared to GFP(-) cells, in Cytoscape using the MCODE algorithm. The clusters were sorted from 1-10 (A-J), based on their individual clustering score. The down-regulated genes are shown in shades of blue, while the up-regulated genes are shown in red. Functional enrichment analysis of the cluster nodes is shown below each cluster, depicting the top 5 terms based on increasing FDR value. The libraries GO Biological Process (non-redundant terms) and Reactome Pathways (non-redundant terms) were used. The grey dashed line corresponds to FDR=0.01.

Figure S8

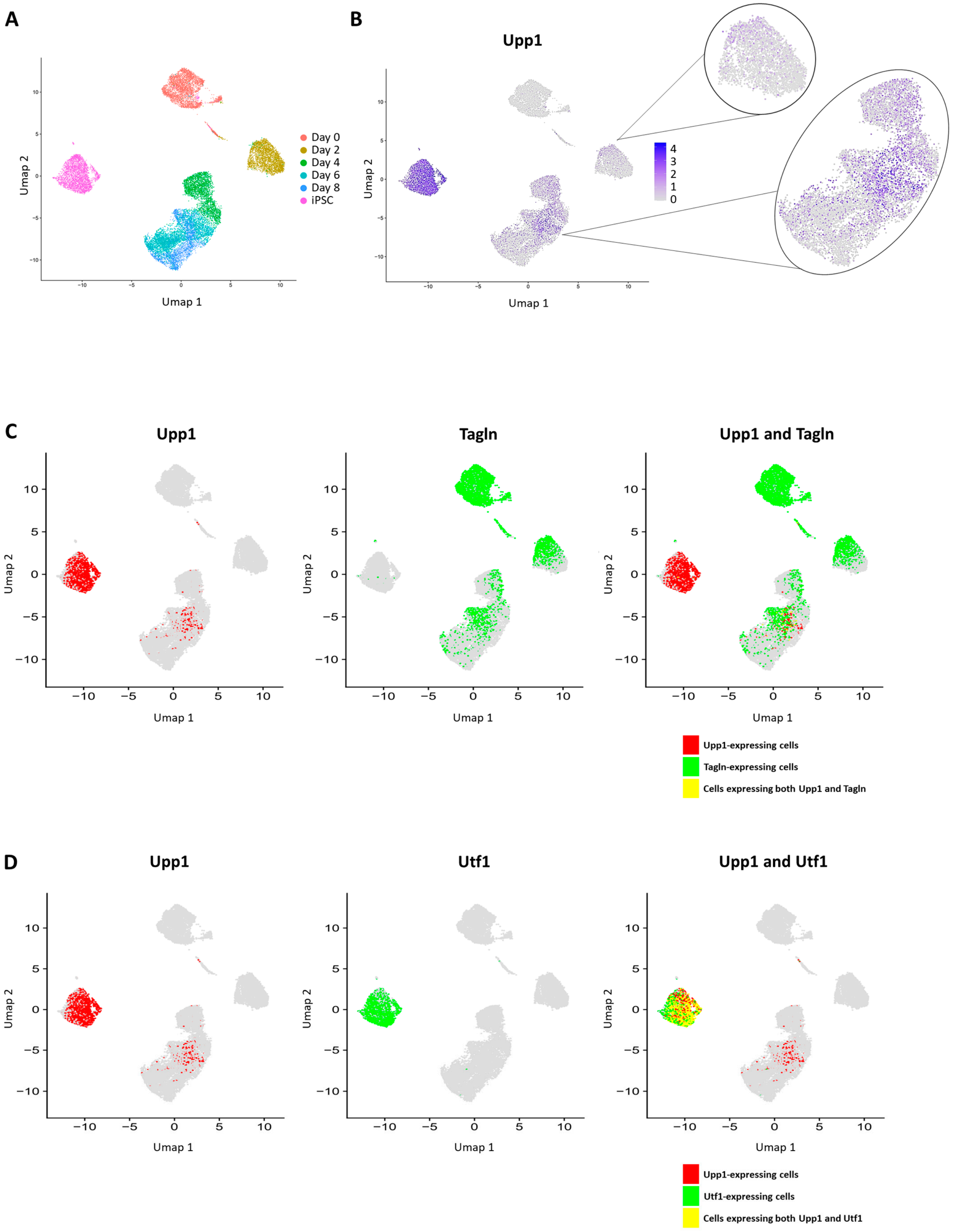

A
B
Upp1
Umap 2
Umap 1
Day 0
Day 2
Day 4
Day 6
Day 8
iPSC
4
3
2
1
0
C
Upp1
Tagln
Upp1 and Tagln
Upp1-expressing cells
Tagln-expressing cells
Cells expressing both Upp1 and Tagln
D
Upp1
Utf1
Upp1 and Utf1
Upp1-expressing cells
Utf1-expressing cells
Cells expressing both Upp1 and Utf1

Figure S8. The expression of the endogenous Upp1 in single-cell resolution. (**A**) Umap analysis of single-cell RNA-seq data during cellular reprogramming (data taken from Schiebinger et al., 2019). Each cell is colored according to the specific day sample (see legend of Figure S8A).; (**B**) As in A, but each cell is colored according to the expression levels of the endogenous *Upp1* gene.; (**C**) As in A, but the cells are colored according to the expression of the endogenous *Upp1* and/or *Tagln* gene. The expression of the *Upp1* gene is represented by red color. The expression of the *Tagln* is shown in green. The simultaneous expression of *Upp1* and *Tagln* is shown in yellow (right panel).; (**D**) As in C, but for the *Upp1* and *Utf1* genes.
